# Steroidal Glycosides from *Convallaria majalis* Whole Plants and Their Cytotoxic Activity

**DOI:** 10.3390/ijms18112358

**Published:** 2017-11-07

**Authors:** Yukiko Matsuo, Daisuke Shinoda, Aina Nakamaru, Kuni Kamohara, Hiroshi Sakagami, Yoshihiro Mimaki

**Affiliations:** 1 School of Pharmacy, Tokyo University of Pharmacy and Life Sciences, 1432-1 Horinouchi, Hachioji, Tokyo 192-0392, Japan; shino.graph617@gmail.com (D.S.); aina_0118@yahoo.co.jp (A.N.); ltstars.lzz@gmail.com (K.K.); mimakiy@toyaku.ac.jp (Y.M.); 2 Research Institute of Odontology, Meikai University; 1-1 Keyaki-dai, Sakado, Saitama 350-0283, Japan; sakagami@dent.meikai.ac.jp

**Keywords:** *Convallaria majalis*, Liliaceae, steroidal glycoside, apoptosis, necrosis, A549, HL-60, HSC-4, HSC-2

## Abstract

Phytochemical examination of *Convallaria majalis* (Liliaceae) whole plants yielded 15 steroidal glycosides (**1**–**15**), including nine new compounds (**4**–**6**, **10**–**15**) with a lycotetrose unit. The structures of the new compounds were determined using two-dimensional Nuclear magnetic resonance (NMR) analyses and chemical methods. The isolated compounds were evaluated for cytotoxicity against HL-60 human promyelocytic leukemia cells, A549 human lung adenocarcinoma cells, and HSC-4 and HSC-2 human oral squamous cell carcinoma cell lines. Of these, (25*S*)-spirost-5-en-3β-yl *O*-β-d-glucopyranosyl-(1→2)-*O*-[β-d-xylopyranosyl-(1→3)]-*O*-β-d-glucopyranosyl-(1→4)-β-d-galactopyranoside (**1**) exhibited cytotoxic activity against HL-60, A549, HSC-4, and HSC-2 cells with IC_50_ values ranging from 0.96 to 3.15 μM. The corresponding furostanol glycoside of 1, (25*S*)-26-[(β-d-glucopyranosyl)oxy]-22α-hydroxyfurost-5-en-3β-yl *O*-β-d-glucopyranosyl-(1→2)-*O*-[β-d-xylopyranosyl-(1→3)]-*O*-β-d-glucopyranosyl-(1→4)-β-d-galactopyranoside (**8**), was cytotoxic to the adherent cell lines of A549, HSC-4, and HSC-2 cells with IC_50_ values of 2.97, 11.04, and 8.25 μM, respectively. The spirostanol lycotetroside (**1**) caused necrotic cell death in A549 cells in a dose-dependent manner. Alternatively, the furostanol lycotetroside (**8**) induced apoptotic cell death in A549 cells in a time-dependent manner, as was evident by morphological observations and flow cytometry analyses.

## 1. Introduction

*Convallaria majalis* L. (Liliaceae), commonly called lily of the valley, is a popular ornamental garden plant [1]. It has been reported to contain cardenolide glycosides, such as convallatoxin and convallatoxol, and is well-known to be a toxic plant [2]. Previously, we conducted phytochemical examinations of the rhizomes of *C. majalis* and isolated and characterized convallasaponin A, a new 5β-spirostanol triglycoside, along with two known cardenolide glycosides, a known cholestane glycoside, and polyhydroxylated steroidal saponins [3,4]. However, thin-layer chromatography (TLC) analysis of the MeOH extract of *C. majalis* indicates that it still contains numerous steroidal glycosides. Our continuing search for steroidal glycosides have revealed that some of them showed potent cytotoxic activity and induced apoptosis against cultured tumor cells. In this study, we conducted further phytochemical analysis of the methanol extract from *C. majalis*, paying particular attention to steroidal glycosides. As a result, nine new steroidal glycosides with an *O*-β-d-glucopyranosyl-(1→2)-*O*-[β-d-xylopyranosyl-(1→3)]-*O*-β-d-glucopyranosyl-(1→4)-β-d-galactopyranose (lycotetrose) unit (**4**–**6**, **10**–**15**) and six known compounds (**1**–**3**, **7**–**9**) were isolated. The structures of the new steroidal glycosides were determined based on spectroscopic analysis, including two-dimensional Nuclear magnetic resonance (NMR) data, and chemical methods. The isolated compounds were evaluated for their cytotoxicity against HL-60 human promyelocytic leukemia cells, A549 human lung adenocarcinoma cells, and HSC-4 and HSC-2 human oral squamous cell carcinoma cells. Apoptosis-inducing activity in A549 cells was also examined.

## 2. Results and Discussion

### 2.1. Structural Elucidation

**Figure 1:**
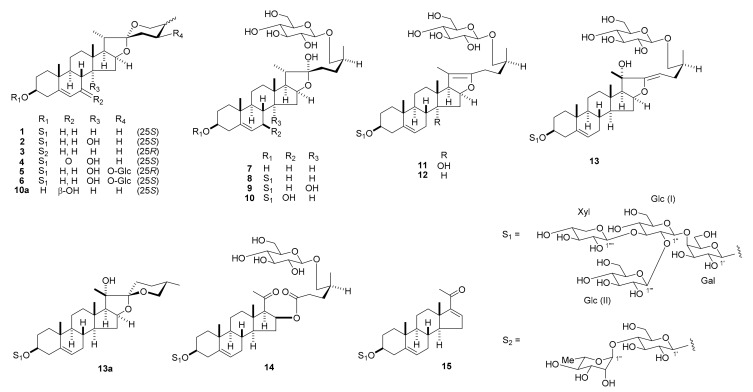
Steroidal glycosides from *Convallaria majalis*.

*Convallaria majalis* (3.0 kg, dry weight) whole plants were extracted with hot MeOH. The MeOH extract was passed through a porous-polymer polystyrene resin (Diaion HP-20, Mitsubishi-Chemical, Tokyo, Japan) column. The MeOH-eluted and MeOH-H_2_O (6:4)-eluted fractions were then subjected to silica gel and octadecylsilanized (ODS) silica gel column chromatography (CC) and reversed-phase preparative high-performance liquid chromatography (HPLC) to obtain compounds **1**–**15** (Figure 1). The structures of the known compounds **1**–**3** and **7**–**9** were identified as (25*S*)-spirost-5-en-3β-yl *O*-β-d-glucopyranosyl-(1→2)-*O*-[β-d-xylopyranosyl-(1→3)]-*O*-β-d-glucopyranosyl-(1→4)-β-d-galactopyranoside (**1**) [5], (25*S*)-14α-hydroxyspirost-5-en-3β-yl O-β-d-glucopyranosyl-(1→2)-*O*-[β-d-xylopyranosyl-(1→3)]-*O*-β-d-glucopyranosyl-(1→4)-β-d-galactopyranoside (**2**) [5], (25*R*)-spirost-5-en-3β-yl *O*-α-L-rhamnopyranosyl-(1→4)-β-d-glucopyranoside (**3**) [6], (25*S*)-26-[(β-d-glucopyranosyl)oxy]-22α-hydroxyfurost-5-en-3β-ol (**7**) [7], (25*S*)-26-[(β-d-glucopyranosyl)oxy]-22α-hydroxyfurost-5-en-3β-yl *O*-β-d-glucopyranosyl-(1→2)-*O*-[β-d-xylopyranosyl-(1→3)]-*O*-β-d-glucopyranosyl-(1→4)-β-d-galactopyranoside (**8**) [8], and (25*S*)-26-[(β-d-glucopyranosyl)oxy]-14α,22α-hydroxyfurost-5-en-3β-yl *O*-β-d-glucopyranosyl-(1→2)-*O*-[β-d-xylopyranosyl-(1→3)]-*O*-β-d-glucopyranosyl-(1→4)-β-d-galactopyranoside (**9**) [9], respectively.

Compound **4** was obtained as an amorphous solid, and its molecular formula was identified as C_50_H_78_O_24_, based on data from high-resolution electrospray ionization time-of-flight mass spectrometry (HR-ESI-TOF-MS; *m*/*z* 1085.4773 [M + Na]^+^, calcd. 1085.4781) and ^13^C NMR (50 carbon signals) spectrum. The ^1^H and ^13^C NMR spectral features of **4** were closely related to those of **2**, showing the following signals: four steroid methyl groups at δ_H_ 1.21 (d, *J* = 7.0 Hz, Me-21), 1.07 (d, *J* = 7.0 Hz, Me-27), 1.04 (s, Me-18), and 0.99 (s, Me-19), in addition to δ_C_ 20.4 (C-18), 17.1 (C-19), 16.3 (C-27), and 15.2 (C-21); an olefinic group at δ_H_ 5.75 (s, H-6) and δ_C_ 166.6 (C-5) and 126.9 (C-6); oxygenated protons and carbons at δ_H_ 3.86 (m, H-3) and δ_C_ 78.1 (C-3), δ_H_ 5.06 (dd, *J* = 12.7, 9.0 Hz, H-16) and δ_C_ 82.2 (C-16), and δ_H_ 4.02 (dd, *J* = 10.9, 2.5 Hz, H-26ax) and 3.31 (br d, *J* = 10.9 Hz, H-26eq) and δ_C_ 65.0 (C-26); an oxygenated quaternary carbon at δ_C_ 110.0 (C-22); and four anomeric protons and carbons at δ_H_ 5.59 (d, *J* = 7.5 Hz), 5.25 (d, *J* = 7.6 Hz), 5.19 (d, *J* = 7.5 Hz), and 4.83 (d, *J* = 7.6 Hz) and δ_C_ 105.2, 105.0, 104.9, and 102.8 (Table 1). In addition, the IR and ^13^C NMR spectra of **4** suggest the presence of a conjugated carbonyl group (ν_max_ 1650 cm^−1^; δ_C_ 200.6) (Table 2). In the heteronuclear multiple-bond correlation (HMBC) spectrum of **4**, long-range correlations observed from H-6 at δ_H_ 5.75, H-9 at δ_H_ 2.41, and H-8 at δ_H_ 2.74 to C-7 at δ_C_ 200.6 indicated that the carbonyl group was located at C-7 of the aglycone. Nuclear Overhauser effect (NOE) correlations were found between the signals of H-8 at δ_H_ 2.74 and Me-18 at δ_H_ 1.04/Me-19 at δ_H_ 0.99, between the signals of H-12ax at δ_H_ 1.41 and H-9 at δ_H_ 2.41/H-17 at δ_H_ 2.72, between the signals of H-17 and Me-21, between the signals of H-20 at δ_H_ 2.02 and Me-18/H-23ax at δ_H_ 1.93, and between the signals of Me-27 at δ_H_ 1.07 and H-23ax at δ_H_ 1.93. These correlations in the NOE spectroscopy (NOESY) spectrum of **4** were consistent with the B/C trans, C/D trans, and D/E cis ring fusions, as well as the 14α, 20α, 22α, and 25*S* configurations. The linkage of lycotetrose, *O*-β-d-glucopyranosyl-(1→2)-*O*-[β-d-xylopyranosyl-(1→3)]-*O*-β-d-glucopyranosyl-(1→4)-β-d-galactopyranose, at C-3 of the aglycone, was ascertained by acid hydrolysis of **4** with 0.5 M HCl, obtaining d-galactose, d-glucose, and d-xylose. Additionally, long-range correlations from the anomeric proton (H-1′′′) of Glc (II) at δ_H_ 5.59 to C-2′′ of Glc (I) at δ_C_ 81.3, from H-1′′′′ of Xyl at δ_H_ 5.25 to C-3′′ of Glc (I) at δ_C_ 86.7, from H-1′′ of Glc (I) at δ_H_ 5.19 to C-4′ of Gal at δ_C_ 79.8, and from H-1′ of Gal at δ_H_ 4.83 to C-3 of the aglycone at δ_C_ 78.1 in the HMBC spectrum of 4 were also observed. Thus, **4** was labeled (25*S*)-3β-[(*O*-β-d-glucopyranosyl-(1→2)-*O*-[β-d-xylopyranosyl-(1→3)]-*O*-β-d-glucopyranosyl-(1→4) β-d-galactopyranosyl)oxy]-14α-hydroxyspirost-5-en-7-one.

The ^1^H and ^13^C NMR spectral data of **5** (C_56_H_90_O_29_) suggest that **5** is a spirostanol glycoside resembling **2** in structure, with a lycotetrose unit at C-3 and a hydroxy group at C-14. However, the molecular formula of **5** was in excess of **2** by C_6_H_10_O_6_, which corresponded to a hexyloxy group. When the ^1^H and ^13^C NMR spectra of **5** were compared with those of **2**, the signals for the C-24 methylene protons at δ_H_ 2.15 and 1.37 (each, m) and carbon at δ_C_ 26.2 were displaced by an oxymethine proton at δ_H_ 4.83 (ddd, *J* = 10.9, 5.5, 5.5 Hz) and carbon at δ_C_ 72.8. Acid hydrolysis of **5** gave d-galactose, d-glucose, and d-xylose as the sugar moieties. Analysis of the ^1^H-^1^H correlation spectroscopy (COSY) and heteronuclear multiple quantum coherence (HMQC) spectra allowed the hexosyl unit attached to C-24 to be assigned β-d-glucopyranosyl, and an HMBC correlation was observed from the anomeric proton at δ_H_ 5.04 (d, *J* = 7.7 Hz) to C-24 carbon at δ_C_ 72.8. In the NOESY spectrum of **5**, NOE correlations between the signals of H-24 at δ_H_ 4.83 and H-25 at δ_H_ 2.24/H-26ax at δ_H_ 3.89, and *J* values of ^3^*J*_H-24, H-23ax_ = 10.9 Hz, ^3^*J*_H-24, H-23eq_ = 5.5 Hz, and ^3^*J*_H-24, H-25_ = 5.5 Hz provided evidence for the 24*S* and 25*R* configurations. Thus, the structure of **5** was determined to be (24*S*,25*R*)-24-[(β-d-glucopyranosyl)oxy]-14α-hydroxyspirost-5-en-3β-yl *O*-β-d-glucopyranosyl-(1→2)-*O*-[β-d-xylopyranosyl-(1→3)]-*O*-β-d-glucopyranosyl-(1→4)-β-d-galactopyranoside.

The molecular formula of **6** (C_56_H_90_O_29_) was the same as that of **5**. The ^1^H and ^13^C NMR spectra of **6** showed close similarity with those of **5**, suggestive of a stereoisomer with respect to the C-25 configuration. In the NOESY spectrum of **6**, the NOE correlations between the signals of H-23ax at δ_H_ 2.00 and H-20 at δ_H_ 2.07/H-25 at δ_H_ 1.91, between the signals of H-26ax at δ_H_ 3.58 and H-16 at δ_H_ 5.05/H-24 at δ_H_ 4.06/Me-27 at δ_H_ 1.13, between the signals of Me-27 and H-24, and *J* values of ^3^*J*_H-24, H-23ax_ = 12.4 Hz, ^3^*J*_H-24, H-23eq_ = 5.3 Hz, and ^3^*J*_H-25, H-26ax_ = 11.8 Hz confirmed the 24*S* and 25*S* configurations. The structure of **6** was characterized as (24*S*,25*S*)-24-[(β-d-glucopyranosyl)oxy]-14α-hydroxyspirost-5-en-3β-yl *O*-β-d-glucopyranosyl-(1→2)-*O*-[β-d-xylopyranosyl-(1→3)]-*O*-β-d-glucopyranosyl-(1→4)-β-d-galactopyranoside.

The hemi-acetal carbon signal at δ_C_ 110.6 and the positive color reaction in Ehrlich’s test suggest that 10 (C_56_H_92_O_29_) may be a 22-hydroxyfurostanol glycoside. The ^1^H and ^13^C NMR spectra of **10** were similar to those of **8**; however, the molecular formula of 10 had one extra oxygen atom compared to that of **8**, and a significant difference was observed in the ^13^C NMR signals from the B-ring portion. Enzymatic hydrolysis of **10** with β-d-glucosidase yielded the corresponding spirostanol saponin **10a**. In the ^1^H-^1^H COSY spectrum of **10a**, a proton signal of a hydroxy group at δ_H_ 5.73 (br d, *J* = 7.8 Hz), which disappeared following the addition of HCl vapor, showed a correlation peak with the C-7 oxymethine proton at δ_H_ 4.01 (m), indicating the presence of a hydroxy group at C-7. The NOE correlations between the signals of H-7 at δ_H_ 4.00 and H-9 at δ_H_ 1.05/H-12ax at δ_H_ 1.68/H-14 at δ_H_ 1.32 in the NOESY spectrum of **10a** confirmed that the C-7 hydroxy group was β-oriented. Furthermore, the NOE correlations between the signals of Me-27 at δ_H_ 1.07 and H-23ax at δ_H_ 1.90 and between the signals of H-25 at δ_H_ 1.57 and H-24ax at δ_H_ 2.14 confirmed the 25*S* configuration. Thus, **10a** was assigned as (25*S*)-7β-hydroxyspirost-5-en-3β-yl *O*-β-d-glucopyranosyl-(1→2)-*O*-[β-d-xylopyranosyl-(1→3)]-*O*-β-d-glucopyranosyl-(1→4)-β-d-galactopyranoside. The linkage of a β-d-glucopyranosyl group (Glc (III)) to the C-26 hydroxy group of the aglycone of **10** was ascertained by an HMBC correlation from H-1′′′′′ of Glc (III) at δ_H_ 4.80 (d, *J* = 7.7 Hz) to C-26 of the aglycone at δ_C_ 75.4. The NOE correlations between the signals of H-20 at δ_H_ 2.24 and H_2_-23 at δ_H_ 2.08 and 1.96 confirmed the C-22α configuration. Therefore, **10** was identified as (25*S*)-26-[(β-d-glucopyranosyl)oxy]-7β,22α-dihydroxyfurost-5-en-3β-yl *O*-β-d-glucopyranosyl-(1→2)-*O*-[β-d-xylopyranosyl-(1→3)]-*O*-β-d-glucopyranosyl-(1→4)-β-d-galactopyranoside.

The ^1^H and ^13^C NMR spectra of **11** (C_56_H_90_O_28_) showed similar features with those of **9**; however, the molecular formula of **11** was smaller than that of **9** by H_2_O, and the signals assignable to H-17 and Me-21 were observed at δ_H_ 3.36 as a doublet (*J* = 9.7 Hz) and δ_H_ 1.67 as a singlet, respectively. Furthermore, signals for a pair of olefinic carbon were detected at δ_C_ 152.2 and 103.9, in addition to those attributable to C-5 and C-6. These data implied that **11** was the corresponding Δ^20(22)^-pseudo-furostanol glycoside of **9**. This was confirmed by the fact that the peracetate (**11a**) of **11** was the same as the product obtained by treating **9** with Ac_2_O in pyridine at 110 °C for 3 h. Enzymatic hydrolysis of **11** with β-d-glucosidase gave the corresponding spirostanol saponin **2** and d-glucose. Accordingly, the structure of **11** was assigned (25*S*)-26-[(β-d-glucopyranosyl)oxy]-14α-hydroxyfurosta-5,20(22)-dien-3β-yl *O*-β-d-glucopyranosyl-(1→2)-*O*-[β-d-xylopyranosyl-(1→3)]-*O*-β-d-glucopyranosyl-(1→4)-β-d-galactopyranoside.

Compound **12** (C_56_H_90_O_27_) appeared to be the corresponding Δ^20(22)^-pseudo-furostanol glycoside of 8 (C_56_H_92_O_28_), based on the characteristic proton signals of the H-17 doublet at δ_H_ 2.44 (*J* = 9.9 Hz) and Me-21 singlet at δ_H_ 1.62, as well as the olefinic carbon signals at δ_C_ 152.4 and 103.5. Enzymatic hydrolysis of **12** with β-d-glucosidase gave the corresponding spirostanol saponin **1** and D-glucose. Thus, the structure of **12** was characterized as (25*S*)-26-[(β-d-glucopyranosyl)oxy]-furosta-5,20(22)-dien-3β-yl *O*-β-d-glucopyranosyl-(1→2)-*O*-[β-d-xylopyranosyl-(1→3)]-*O*-β-d-glucopyranosyl-(1→4)-β-d-galactopyranoside.

**Figure 2:**
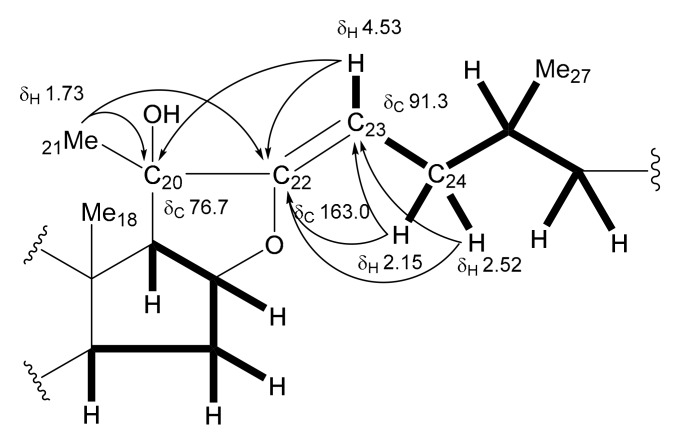
Heteronuclear multiple-bond correlation (HMBC) correlations of **13**. Bold lines indicate the ^1^H-^1^H couplings and arrows indicate ^1^H/^13^C long-range correlations.

**Figure 3:**
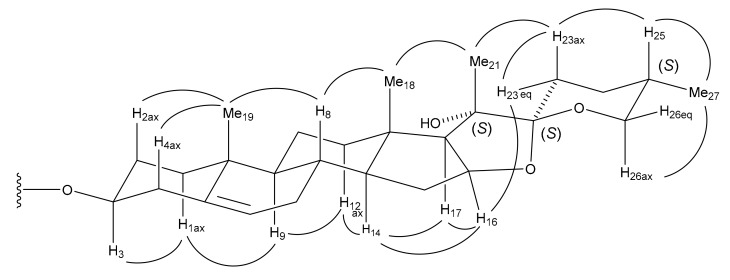
Key Nuclear Overhauser effect (NOE) correlations of **13a**.

The ^1^H and ^13^C NMR spectral data for **13** (C_56_H_90_O_28_) suggest that it is a furostanol glycoside, having a structure similar to that of **8**. However, significant differences were recognized in the signals from the ring E and the side chain moiety, where a tertiary hydroxy group [δ_C_ 76.7 (C)] and an oxygen-bearing trisubstituted olefinic group [δ_C_ 163.0 (-O-C(C)=CH-); δ_C_ 91.3 (-O-C(C)=CH-)/δ_H_ 4.53 (br d, *J* = 13.8 Hz)] were supposed to be located. The deshielded methyl singlet signal at δ_H_ 1.73 assignable to Me-21 and the olefinic proton at δ_H_ 4.53 exhibited long-range correlations with the quaternary carbon at δ_C_ 76.7 and the oxygen-bearing olefinic carbon at δ_C_ 163.0 in the HMBC spectrum of **13**. The olefinic proton at δ_H_ 4.53 was shown to be coupled with the methylene protons at δ_H_ 2.52 and 2.15 (each, m) attributable to H_2_-24 in the ^1^H-^1^H COSY spectrum. These correlations allowed the tertiary hydroxy group and trisubstituted olefinic group to be placed at C-20 and between C-22 and C-23, respectively. The other correlations supporting the partial structure are depicted in Figure 2. The geometry of the olefinic group was determined to be Z by an NOE correlation between the signals of Me-21 at δ_H_ 1.73 and H-23 at δ_H_ 4.53. Enzymatic hydrolysis of **13** with β-d-glucosidase yielded the spirostanol saponin **13a** and D-glucose. The NOE correlations between the signals of Me-21 at δ_H_ 1.73 and Me-18 at δ_H_ 1.19/H-23ax at δ_H_ 1.79, between the signals of H-23eq at δ_H_ 2.37 and H-16 at δ_H_ 5.06, and between the signals of H-25 at δ_H_ 1.69 and H-23ax/Me-27 at δ_H_ 0.75 in the NOESY spectrum of **13a**, in addition to the large *J* value of the H-26ax proton (^3^*J*_H-26ax, H-25_ = 10.0 Hz), were indicative of the 20*S*, 22*S*, and 25*S* configurations (Figure 3). Thus, spirostanol saponin **13a** was identified as (20*S*,22*S*,25*S*)-20-hydroxyspirost-5-en-3β-yl *O*-β-d-glucopyranosyl-(1→2)-*O*-[β-d-xylopyranosyl-(1→3)]-*O*-β-d-glucopyranosyl-(1→4)-β-d-galactopyranoside. An HMBC correlation from H-1′′′′′ of Glc (III) at δ_H_ 4.85 (d, *J* = 7.7 Hz) to C-26 of the aglycone at δ_C_ 75.3 in the HMBC spectrum of **13** confirmed that a β-d-glucopyranosyl group was attached to C-26. Therefore, the structure of **13** was elucidated to be (20*S*,22*Z*,25*S*)-26-[(β-d-glucopyranosyl)oxy]-20-hydroxyfurosta-5,22-dien-3β-yl *O*-β-d-glucopyranosyl-(1→2)-*O*-[β-d-xylopyranosyl-(1→3)]-*O*-β-d-glucopyranosyl-(1→4)-β-d-galactopyranoside.

The ^1^H and ^13^C NMR data for **14** (C_56_H_90_O_29_) were essentially analogous to those of **12**, except for the lack of signals for the C-20(22)-tetrasubstituted olefinic group at δ_C_ 103.5 and 152.4. Instead, signals for a keto carbonyl carbon at δ_C_ 205.5 and an ester carbonyl carbon at δ_C_ 173.2 were newly observed in the ^13^C NMR spectrum of **14**. All other signals appeared at almost the same positions between the two glycosides. In the HMBC spectrum of **14**, the H-17 at δ_H_ 2.48 (d, *J* = 7.6 Hz) and Me-21 protons at δ_H_ 2.13 (s) showed long-range correlations with the keto carbonyl carbon at δ_C_ 205.5, which was assigned to C-20. Long-range correlations from the H-16 at δ_H_ 5.66 (m) and H_2_-23 protons at δ_H_ 1.84 (m) and 1.47 (m) to the ester carbonyl carbon at δ_C_ 173.2 resulted in the assignment of the ester carbonyl carbon to C-22. These data suggest that **14** was formed from **12** through the oxidative cleavage of the C-20(22) double bond. This was confirmed by the fact that the peracetate (**14a**) of **14** was identical to the product obtained by treating **12** with Ac_2_O in pyridine at room temperature for 12 h and then with CrO_3_ in AcOH. Accordingly, the structure of **14** was determined to be 16β-[[(4*S*)-5-(β-d-glucopyranosyloxy)-4-methyl-1-oxo-pentyl]oxy]-3β-[(*O*-β-d-glucopyranosyl-(1→2)-*O*-[β-d-xylopyranosyl-(1→3)]-*O-*β-d-glucopyranosyl-(1→4)-β-d-galactopyranosyl)oxy]-pregn-5-en-20-one.

The data suggest that **15** (C_44_H_68_O_21_) is a pregnane glycoside. Its ^1^H NMR spectrum showed signals for two angular methyl groups at δ_H_ 0.90 (s, Me-18) and 0.86 (s, Me-19), a methyl group of an acetyl moiety at δ_H_ 2.22 (s, Me-21), two olefinic protons at δ_H_ 6.57 (br s, H-16) and 5.29 (br d, *J* = 5.4 Hz, H-6), and four anomeric protons at δ_H_ 5.58 (d, *J* = 7.8 Hz, H-1′′′), 5.23 (d, *J* = 7.8 Hz, H-1′′′′), 5.18 (d, *J* = 7.8 Hz, H-1′′), and 4.88 (d, *J* = 7.8 Hz, H-1′). The existence of an α,β-unsaturated carbonyl group was verified by the IR (1660 cm^−1^), UV [239 nm (logε 3.81)], and ^13^C NMR [δ_C_ 196.1 (C=O), 155.0 (C), and 144.5 (CH)] spectra. These spectral data and comparison with those of previously reported compounds identified the aglycone of **15** as 3β-hydroxypregna-5,16-dien-20-one [10]. Acid hydrolysis of **15** and analysis of HMBC correlations provided evidence that a lycotetrose was attached to C-3 of the aglycone. Thus, the structure of **15** was assigned 3β-[(*O*-β-d-glucopyranosyl-(1→2)-*O*-[β-d-xylopyranosyl-(1→3)]-*O*-β-d-glucopyranosyl-(1→4)-β-d-galactopyranosyl)oxy]-pregna-5,16-dien-20-one.

### 2.2. Cytotoxic Activity

The isolated compounds **1**–**15** were evaluated for their cytotoxic activity against HL-60 human promyelocytic leukemia cells, A549 human lung adenocarcinoma cells, HSC-4, and HSC-2 human oral squamous cell carcinoma cells. Etoposide, cisplatin, and doxorubicin were used as positive controls. Compound **1** showed cytotoxic activity against the four tumor cell lines with IC_50_ values ranging from 0.96 ± 0.01 to 3.15 ± 0.43 μM. Compound **8**, which is the corresponding furostanol glycoside of **1**, was only cytotoxic to the adherent cell lines of A549, HSC-4, and HSC-2 cells, with IC_50_ values of 2.97 ± 0.06 μM, 11.04 ± 0.25 μM, and 8.25 ± 0.20 μM, respectively (Table 3). As we previously reported [11,12], the cytotoxicity of **1** compared to those of **2**, **4**, **5**, and **6** and of **8** compared to those of **9** and **10** indicated that the introduction of polar substituents to the steroidal nuclei resulted in reduced the cytotoxicity. Compound **1** was cytotoxic to tumor cells, whereas **3**, having the diglycoside did not show cytotoxic activity. These results implied that not only the structures of the aglycone moiety but also the sugar sequences in the steroidal glycosides considerably contributed to the appearance of cytotoxicity. In previous study, furostanol and pseudo-furostanol glycosides were generally inactive, and on the other hand, 3-*O*-lycotetroside showed significant cytotoxic activities [13]. In this study, correlation between the structure and cytotoxicity of steroidal glycosides have provided similar results. Isolated compounds were evaluated for their cytotoxic activity against TIG-3 normal human diploid fibroblasts. As a result, unfortunately, **1** and **8** gave IC_50_ values of 1.30 ± 0.72 and 3.27 ± 0.13 µM, respectively. However, cisplatin, a clinically used anticancer agent, was also toxic to healthy cells TIG-3 with IC_50_ value of 7.64 ± 0.08 μM in vitro evaluation.

**Figure 4:**
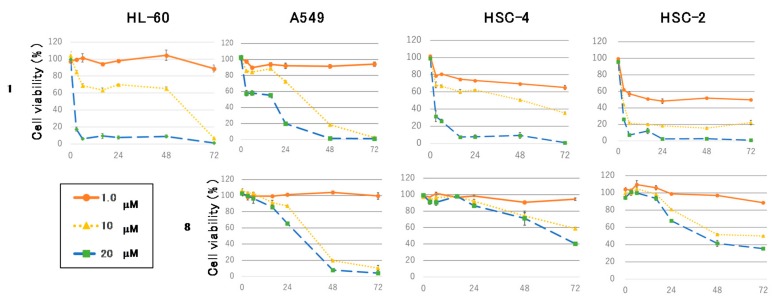
Cell viability of HL-60, A549, HSC-4 and HSC-2 cells after treatment with 1.0, 10, and 20 μM of **1** and **8** for 0, 3, 6, 16, 24, 48, and 72 h. Data are represented as the mean value ± SEM of three experiments performed in triplicate.

**Figure 5:**
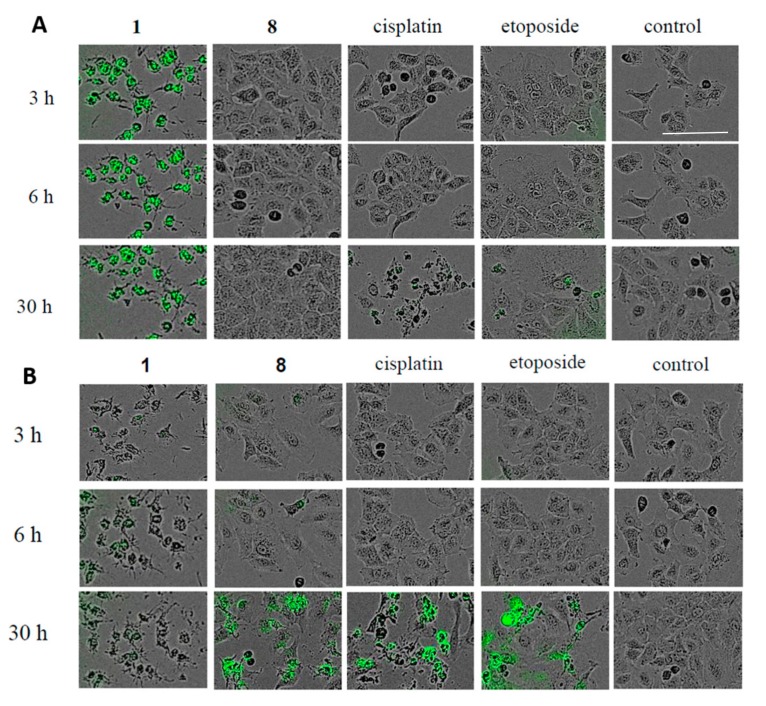
(**A**) Phase-contrast and fluorescent images of A549 cells stained with Cell Player^TM^ 96-well caspase-3/7 Apoptosis Assay kit, showing morphological changes in response to **1** (20 μM), **8** (20 μM), cisplatin (33 μM), and etoposide (33 μM). (**B**) Phase-contrast and fluorescent images of A549 cells stained with YOYO-1, showing morphological changes in response to **1** (20 μM), **8** (20 μM), cisplatin (33 μM), and etoposide (33 μM). Scale bar = 100 μM.

Figure 4 shows the time course of the antiproliferative effects of **1** or **8** at 1.0, 10, and 20 μM on HL-60, A549, HSC-4, and HSC-2 cells. Compound **1** decreased tumor cells viability in a dose-dependent manner within 16 h. In contrast, **8** reduced adherent cells viability in a time-dependent manner. HL-60, A549, HSC-4, and HSC-2 cells were exposed to **1** or **8** and then stained with 4′,6-diamidino-2-phenylindole dihydrochloride (DAPI). Treatment of the cells with **1** led to necrotic cell death in the four cell lines, whereas the cells exposed to **8** morphologically displayed nuclear chromatin condensation and nuclear disassembly, implying that 8 induced apoptosis in the adherent cells (Supplementary Material 1). Next, the activation of caspase-3/7 in A549 cells treated with **1** or **8** was evaluated using the Cell Player^TM^ caspase-3/7 Apoptosis Assay kit. When A549 cells were treated with 20 μM of **8** for 30 h, a significant green fluorescence was detected (Figure 5A), indicating that **8** induced apoptotic cell death in A549 cells through the activation of caspase-3/7. Alternatively, phase-contrast and fluorescent images of the A549 cells treated with 20 μM of **1** followed by staining with oxazole yellow dimer (YOYO-1), which stains the nuclear DNA in permeabilized cells [14], showed that the cell membrane became permeable at an early stage of the necrotic cell death process (with green fluorescence) (Figure 5B). Finally, the cell cycle distribution of A549 cells treated with **8** for 30 h was analyzed using flow cytometry. Compound 8 increased the sub-G1 cell population from 4.07% to 14.10% in A549 cells (Table 4). These results suggest that **8** induced apoptotic death in A549 cells through caspase-3/7 activity in a time-dependent manner, whereas **1** induced necrotic cell death in A549 cells.

## 3. Experimental Section

### 3.1. General

Optical rotations were measured using a JASCO P-1030 (JASCO, Tokyo, Japan). UV spectra were recorded on a JASCO V-630. IR spectra were recorded on a JASCO FT-IR 410. ^1^H NMR spectra were recorded with a DRX-500 spectrometer (Bruker, Karlsruhe, Germany) using standard Bruker pulse programs at 300 K. Chemical shifts are given as δ values relative to tetramethylsilane (TMS), which was used as an internal standard. HR-ESI-TOF-MS data were recorded on the LCT mass spectrometer (Waters-Micromass, Manchester, UK). Diaion HP-20 (50 mesh; Mitsubishi-Chemical, Tokyo, Japan), BW-300 silica gel (200-300 mesh; Fuji Silysia Chemical, Kasugai, Japan), and ODS silica gel COSMOSIL 75C_18_-OPN (75 μM; Nacalai Tesque, Kyoto, Japan) were used for CC. TLC was carried out on precoated silica gel 60 F_254_ (0.25 mm thick; Merck, Darmstadt, Germany) and RP_18_ F_254_S plates (0.25 mm thick; Merck), and the spots were visualized by spraying the plates with 10% H_2_SO_4_ (aq) followed by heating. HPLC was performed using a system composed of a CCPM pump (Tosoh, Tokyo, Japan), a CCP PX-8010 controller (Tosoh), an RI-8010 detector (Tosoh), and a Rheodyne injection port (Rheodyne LLC, Rohnert Park, CA, USA). A TSK gel ODS-100Z column (10 mm i.d. × 250 mm, 5 μm; Tosoh) was employed for preparative HPLC. The purities of all the isolated compounds were confirmed by their ^1^H and ^13^C NMR spectra. The following materials and biochemical-grade reagents were used for the cell cultures and the cytotoxicity assays: a microplate reader (Spectra Classic, Tecan, Salzburg, Austria); a 96-well flat-bottom plate (Iwaki Glass, Funabashi, Japan); JCRB 0085 HL-60 and JCRB 0076 A549 cells (Human Science Research Resources Bank, Osaka, Japan); HSC-2 and HSC-4 cells (Riken Cell Bank, Tsukuba, Japan); fetal bovine serum (FBS; Nichirei Biosciences, Tokyo, Japan); 0.25% trypsin-ethylenediaminetetraacetic acid (EDTA) solution, RPMI-1640 medium, minimum essential medium (MEM), etoposide, Triton X-100, and 3-(4,5-dimethylthiazol-2-yl)-2,5-diphenyl-2H-tetrazolium bromide (MTT) (Sigma-Aldrich, St. Louis, MO, USA); penicillin G sodium salt and streptomycin sulfate (Gibco, Grand Island, NY, USA); paraformaldehyde and ribonuclease A (Wako Pure Chemical Industries, Osaka, Japan); and propidium iodide (PI) (Molecular Probes, Eugene, OR, USA).

### 3.2. Isolation and Structural Determination

#### 3.2.1. Plant Material

*C. majalis* whole plants (dry weight, 3.0 kg) were obtained from Richters Co., Ltd. (Goodwood, ON, Canada). A voucher specimen was deposited at the Herbarium of the Tokyo University of Pharmacy and Life Sciences (KS-2011-004).

#### 3.2.2. Extraction and Isolation

*C. majalis* whole plants (3.0 kg, dry weight) were extracted with MeOH under reflux for 4 h. After removing the solvent, the MeOH extract (460 g) was passed through a Diaion HP-20 column (2000 g, 8.5 cm i.d. × 60 cm) and successively eluted with MeOH-H_2_O (3:7, 6:4), MeOH, EtOH, and EtOAc (6 L of each eluent). CC of the MeOH-eluted fraction (100 g) on silica gel (2000 g, 8.0 cm i.d. × 40 cm), eluted with a stepwise gradient mixture of CHCl_3_-MeOH-H_2_O (9:1:0, 40:10:1, 20:10:1, 7:4:1) and finally with MeOH, provided 9 fractions (Frs. M-1 to M-9). Fr. M-3 was chromatographed on ODS silica gel (800 g, 6.0 cm i.d. × 25 cm) eluted with MeOH-H_2_O (1:1, 2:1, 3:1, 4:1) and finally with MeOH, providing 12 subfractions (Frs. M-3-1 to M-3-12). Fr. M-3-11 was separated by HPLC (1.0 cm i.d. × 25 cm) using MeCN-H_2_O (2:3) to isolate **3** (2.8 mg) and **7** (14.0 mg). Fr. M-6 was chromatographed on ODS silica gel (800 g, 6.0 cm i.d. × 30 cm) eluted with MeOH-H_2_O (2:1, 3:1, 4:1) to yield **1** (22.3 mg), **2** (68.7 mg), and **4** (5.1 mg). Fr. M-7 (94.0 mg) was separated by a silica gel column (1500 g, 6.0 cm i.d. × 30 cm) eluted with CHCl_3_-MeOH-H_2_O (7:4:1) and an ODS silica gel column (800 g, 6.0 cm i.d. × 28 cm) eluted with MeOH-H_2_O (3:2, 3:1) to yield **12** (13.7 mg), **13** (21.4 mg), **14** (26.4 mg), and **15** (14.5 mg). CC of the MeOH-H_2_O (6:4)-eluted fraction (40 g) on silica gel (1500 g, 7.5 cm i.d. × 40 cm), eluted with a stepwise gradient mixture of CHCl_3_-MeOH-H_2_O (7:4:1) and finally with MeOH, provided 8 fractions (Frs. MH-1 to MH-8). Fr. MH-7 (9.6 g) was chromatographed on ODS silica gel (800 g, 6.0 cm i.d. × 28 cm) eluted with MeOH-H_2_O (1:1, 2:1) and finally with MeOH, providing 9 subfractions (Frs. MH-7-1 to MH-7-9). Fr. MH-7-3 (487 mg) was separated by HPLC (1.0 cm i.d. × 25 cm) using MeCN-H_2_O (1:3) to isolate **5** (16.9 mg), **6** (11.9 mg), and **9** (98.3 mg). Fr. MH-7-6 (640 mg) was separated by ODS silica gel CC (500g, 4.0 cm i.d. × 30 cm) eluted with MeCN-H_2_O (1:4) to yield **8** (395 mg), **10** (20.9 mg), and **11** (20.3 mg).

#### 3.2.3. Structural Characterization

Compound **4**: An amorphous solid. [α]_D_^25^ –50.4 (*c* 0.25, MeOH). HR-ESI-TOF-MS *m/z*: 1085.4773 [M + Na]^+^ (calcd for C_50_H_78_NaO_24_: 1085.4781). IR ν_max_ (film) cm^−1^: 3152 (OH), 2948 (CH), 1650 (C=O). UV λ_max_ (MeOH) nm (log ε): 237 (3.86). For ^1^H and ^13^C NMR spectral data, see Supplementary Materials 1 and 2. For ^1^H and ^13^C NMR spectral data of the sugar moiety, see Table 1. For ^13^C-NMR spectral data of the aglycone moiety, see Table 2.

Compound **5**: An amorphous solid. [α]_D_^25^ –64.6 (*c* 0.14, MeOH). HR-ESI-TOF-MS *m/z*: 1249.5472 [M + Na]^+^ (calcd for C_56_H_90_NaO_29_: 1249.5465). IR ν_max_ (film) cm^−1^: 3420 (OH), 2930 (CH). For ^1^H and ^13^C NMR spectral data, see Supplementary Materials 1 and 2. For ^13^C-NMR spectral data of the aglycone moiety, see Table 2.

Compound **6**: An amorphous solid. [α]_D_^25^ –38.2 (*c* 0.08, MeOH). HR-ESI-TOF-MS *m/z*: 1249.5477 [M + Na]^+^ (calcd for C_56_H_90_NaO_29_: 1249.5465). IR ν_max_ (film) cm^−1^: 3365 (OH), 2929 (CH). For ^1^H and ^13^C NMR spectral data, see Supplementary Materials 1 and 2. For ^13^C-NMR spectral data of the aglycone moiety, see Table 2.

Compound **10**: An amorphous solid. [α]_D_^25^ –49.4 (*c* 0.14, MeOH). HR-ESI-TOF-MS *m/z*: 1251.5625 [M + Na]^+^ (calcd for C_56_H_92_NaO_29_: 1251.5622). IR ν_max_ (film) cm^−1^: 3398 (OH), 2874 (CH). For ^1^H and ^13^C NMR spectral data, see Supplementary Materials 1 and 2. For ^13^C-NMR spectral data of the aglycone moiety, see Table 2.

Compound **11**: An amorphous solid. [α]_D_^25^ –29.1 (*c* 0.10, MeOH). HR-ESI-TOF-MS *m/z*: 1233.5516 [M + Na]^+^ (calcd for C_56_H_90_NaO_28_: 1233.5516). IR ν_max_ (film) cm^−1^: 3397 (OH), 2924 (CH). For ^1^H and ^13^C NMR spectral data, see Supplementary Materials 1 and 2. For ^13^C-NMR spectral data of the aglycone moiety, see Table 2.

Compound **12**: An amorphous solid. [α]_D_^25^ –33.3 (*c* 0.18, MeOH). HR-ESI-TOF-MS *m/z*: 1195.5739 [M + H]^+^ (calcd for C_56_H_91_O_27_: 1195.5748). IR ν_max_ (film) cm^−1^: 3397 (OH), 2935 (CH). For ^1^H and ^13^C NMR spectral data, see Supplementary Materials 1 and 2. For ^13^C-NMR spectral data of the aglycone moiety, see Table 2.

Compound **13**: An amorphous solid. [α]_D_^25^ –38.1 (*c* 0.09, MeOH). HR-ESI-TOF-MS *m/z*: 1233.5531 [M + Na]^+^ (calcd for C_56_H_90_NaO_28_: 1233.5516). IR ν_max_ (film) cm^−1^: 3366 (OH), 2931 and 2872 (CH). For ^1^H and ^13^C NMR spectral data, see Supplementary Materials 1 and 2. For ^13^C-NMR spectral data of the aglycone moiety, see Table 2.

Compound **14**: An amorphous solid. [α]_D_^25^ –37.9 (*c* 0.18, MeOH). HR-ESI-TOF-MS *m/z*: 1249.5466 [M + Na]^+^ (calcd for C_56_H_90_NaO_29_: 1249.5465). IR ν_max_ (film) cm^−1^: 3447, 3423, and 3400 (OH), 2923 and 2883 (CH), 1731 and 1707 (C=O). For ^1^H and ^13^C NMR spectral data, see Supplementary Materials 1 and 2. For ^13^C-NMR spectral data of the aglycone moiety, see Table 2.

Compound **15**: An amorphous solid. [α]_D_^25^ –32.8 (*c* 0.10, MeOH). HR-ESI-TOF-MS *m/z*: 955.4127 [M + Na]^+^ (calcd for C_44_H_68_NaO_21_: 955.4151). IR ν_max_ (film) cm^−1^: 3481 (OH), 2921 and 2889 (CH), 1660 (C=O). UV λ_max_ (MeOH) nm (log ε): 239 (3.81). For ^1^H and ^13^C NMR spectral data, see Supplementary Materials 1 and 2. For ^13^C-NMR spectral data of the aglycone moiety, see Table 2.

##### Acid Hydrolysis of **4**–**6**, **10a**, **13a**, and **15**

A solution of **4** (3.0 mg) in 0.5 M HCl and dioxane-H_2_O (1:1; 2.0 mL) was heated at 95 °C for 2 h under an Ar atmosphere. The reaction mixture was neturalized by passing it through an Amberlite IRA-96SB column (16–50 mesh, 50 g, 1.5 cm i.d. × 15 cm; Organo, Tokyo, Japan). The mixture was then eluted through a Diaion HP-20 column (50 g, 1.5 cm i.d. × 15 cm) with MeOH-H_2_O (4:6) and EtOH-Me_2_CO (1:1). The sugar fraction was analyzed using HPLC under the following conditions: Capcell Pak NH2 UG80 column (4.6 mm i.d. × 25 cm, 5 μm; Shiseido, Tokyo, Japan); mobile phase of MeCN-H_2_O (85:15); detection by refractive index and optical rotation; and a flow rate of 1.0 mL/min. d-Glucose, d-galacose, and d-xylose were identified by comparing their retention times (*t*_R_) and optical rotation with those of authentic samples: d-galactose (12.66, positive optical rotation), d-glucose (14.34, positive optical rotation), and d-xylose (9.21, positive optical rotation). Compounds **5** (5.0 mg), **6** (3.5 mg), **10a** (3.5 mg), **13a** (3.0 mg), and **15** (2.0 mg) were independently subjected to acid hydrolysis as described for **4**. HPLC analysis of the sugar fractions under the same conditions as in the case of **4** indicated the presence of d-galacose, d-glucose, and d-xylose.

##### Enzymatic Hydrolysis of **10**, **11**, **12**, and **13**

Compound **10** (10.0 mg) was treated with β-d-glucosidase (15.0 mg, EC 232-589-7; Sigma-Aldrich) in HOAc/NaOAc buffer (pH 5.0, 3.0 mL) at room temperature for 12 h. The reaction mixture was purified using CC on silica gel (100 g, 2 cm i.d. × 30 cm) eluted with CHCl_3_-MeOH-H_2_O (20:10:1) to obtain gitogenin (**10a**, 4.2 mg) and a sugar fraction. HPLC analysis of the sugar fraction under the same conditions as those used for **4** indicated the presence of d-glucose (14.28, positive optical rotation). Compounds **11** (5.0 mg), **12** (5.0 mg), and **13** (7.0 mg) were independently subjected to enzymatic hydrolysis as described for **10** to obtain **2** (2.0 mg; from **11**), **1** (1.0 mg; from **12**), **13a** (3.3 mg; from **13**), and sugar fractions. HPLC analysis of the sugar fractions under the same conditions as those used for **4** indicated the presence of d-glucose (**11**: 13.98, positive optical rotation; **12**: 14.07, positive optical rotation; **13**: 14.11, positive optical rotation).

##### Acetylation of **11** and **14**

Compounds **11** (2.0 mg) and **14** (3.6 mg) were independently acetylated with Ac_2_O (1.0 mL) in pyridine (1.0 mL) at room temperature for 18 h. Each crude acetate was purified using preparative TLC with hexane-Me_2_CO (1:1) to yield **11a** (1.9 mg) and **14a** (2.8 mg).

##### Preparation of **11a** from **9**

A mixture of **9** (15.0 mg) and Ac_2_O (5.0 mL) in pyridine (3.0 mL) was stirred at 110 °C for 3 h. After the excess Ac_2_O was decomposed by H_2_O (10 mL), the reaction mixture was purified using preparative TLC with hexane-Me_2_CO (7:5) to yield **11a** (4.5 mg).

##### Preparation of **14a** from **12**

Compound **12** (5.0 mg) was treated with Ac_2_O (2.0 mL) in pyridine (4.0 mL) at room temperature for 12 h. After the excess Ac_2_O was decomposed by H_2_O (10 mL), the reaction mixture was evaporated to dryness. The crude product was dissolved in 95% AcOH (aq.) (5.0 mL), to which the CrO_3_ (15.0 mg) solution in 95% AcOH (aq) (1.0 mL) was added, and the mixture was stirred at room temperature for 3 h. After the excess CrO_3_ was decomposed by MeOH (3.0 mL), the crude product was diluted with H_2_O (20.0 mL) and extracted with Et_2_O (20.0 mL × 3). The Et_2_O extract was purified using ODS Si CC (100 g, 3 cm i.d. × 25 cm) eluted with MeCN-H_2_O (3:1) to obtain **14a** (8.2 mg).

Compound **10a**: An amorphous solid. [α]_D_^25^ –21.7 (*c* 0.09, MeOH). HR-ESI-TOF-MS *m/z*: 1071.4994 [M + Na]^+^ (calcd for C_50_H_80_NaO_23_: 1071.4988). IR ν_max_ (film) cm^−1^: 3335 (OH), 2928 (CH). For ^1^H and ^13^C NMR spectral data, see Supplementary Materials 1 and 2.

Compound **11a**: An amorphous solid. [α]_D_^25^ –14.3 (*c* 0.05, MeOH). HR-ESI-TOF-MS *m/z*: 1925.7460 [M + H]^+^ (calcd for C_90_H_125_O_45_: 1925.7493). IR ν_max_ (film) cm^−1^: 2924 and 2854 (CH), 1748 (C=O). ^1^H NMR (500 MHz, C_5_D_5_N): δ_H_ 5.65 (1H, br d, *J* = 1.9 Hz, H-6), 3.97 (1H, dd, *J* = 9.5, 5.3 Hz, H-26a), 3.70 (1H, m, *W*_1/2_ = 29.1 Hz, H-3), 3.45 (1H, dd, *J* = 9.5, 6.3 Hz, H-26b), 2.38, 2.34, 2.32, 2.16 × 2, 2.14, 2.13 × 2, 2.07, 2.04 × 2, 2.03, 2.01, 1.97 × 2, 1.96 (each 3H, s, Ac × 16), 1.69 (3H, s, Me-21), 1.15 (3H, s, Me-18 or Me-19), 0.98 (3H, d, *J* = 6.6 Hz, Me-27), 0.97 (3H, s, Me-18 or Me-19).

Compound **13a**: An amorphous solid. [α]_D_^25^ –22.5 (*c* 0.15, MeOH). HR-ESI-TOF-MS *m/z*: 1071.4935 [M + Na]^+^ (calcd for C_50_H_80_NaO_23_: 1071.4988). IR ν_max_ (film) cm^−1^: 3328 (OH), 2926 (CH). For ^1^H and ^13^C NMR spectral data, see Supplementary Materials 1 and 2.

Compound **14a**: An amorphous solid. [α]_D_^25^ –21.2 (*c* 0.14, MeOH). HR-ESI-TOF-MS *m/z*: 1921.7156 [M + Na]^+^ (calcd for C_88_H_122_NaO_45_: 1921.7156). IR ν_max_ (film) cm^−1^: 2936 (CH), 1750 (C=O). ^1^H NMR (500 MHz, C_5_D_5_N): δ_H_ 3.72 (1H, m, *W*_1/2_ = 28.0 Hz, H-3), 3.40 (1H, dd, *J* = 9.9, 6.4, H-26a), 2.39, 2.35, 2.33 (each 3H, s, Ac × 3), 2.17―2.13 (Ac × 5), 2.07 × 2, 2.05, 2.03, 2.02, 1.98, 1.97, 1.96 (each 3H, s, Ac × 8), 1.23 (3H, s, Me-18 or Me-19), 1.04 (3H, s, Me-18 or Me-19), 0.90 (3H, d, *J* = 6.5 Hz, Me-27).

### 3.3. Biological Activity

#### 3.3.1. Cell culture and Cytotoxicity Assays

HL-60 cells were maintained in an RPMI-1640 medium, A549 and TIG-3 cells were maintained in MEM, and HSC-4 and HSC-2 cells were maintained in DMEM. The cell media contained heat-inactivated 10% (*v*/*v*) FBS supplemented with L-glutamine, penicillin G sodium salt (100 units/mL), and streptomycin sulfate (100 μg/mL). HL-60 (4 × 10^4^ cells/mL), A549 (1 × 10^4^ cells/mL), HSC-4 (2 × 10^4^ cells/mL), HSC-2 (1 × 10^4^ cells/mL), and TIG-3 (5 × 10^4^ cells/mL) cells were continuously treated with each compound for 72 h, and cell growth was measured using an MTT reduction assay as previously described [15]. Data are represented as mean ± standard error of the mean (SEM) of three experiments performed in triplicate. The concentration, up to 12 μM, resulting in a 50% inhibition value (IC_50_) was calculated from the dose response curve. The time courses for 0, 3, 6, 16, 24, 48, and 72 h of the antiproliferative effects of **1** and **8** at 1.0, 10, and 20 μM on HL-60, A549, HSC-4, and HSC-2 cells were also examined using the MTT assay.

#### 3.3.2. Cell Morphology Analysis

HL-60 (2 × 10^4^ cells/well), A549 (1 × 10^4^ cells/well), HSC-4 (1 × 10^4^ cells/well) and HSC-2 (4 × 10^3^ cells/well) were independently plated on coverslips in 96-well plates. After 24 h, the cells were treated with either **1** (20 μM) for 3 h, **8** (20 μM) for 24 h, etoposide (15 μM), or cisplatin (33 μM). The cells were fixed with 1% glutaraldehyde for 30 min at room temperature and before staining with DAPI (0.5 μg/mL). They were observed using a CKX41 fluorescence microscope (Olympus, Tokyo, Japan). A549 cells (1 × 10^5^ cells/well) were seeded in 96-well plates. After 24 h, the A549 cells were treated with either **1** (20 μM) or **8** (20 μM) and stained with caspase-3/7 assay reagent (1 μM) (Essen BioScience, Hertfordshire, UK) or YOYO-1 (1 μM; Invitrogen, Carlsbad, CA, USA). Cells images were obtained after 3, 6, and 30 h, respectively, using the IncuCyte^®^ ZOOM (Essen BioScience, Hertfordshire, UK) in 5% CO_2_/air at 37 °C.

#### 3.3.3. Cell Cycle Analysis by Flow Cytometry

Cell cycle distribution was evaluated using a flow cytometer (FACSCanto II, BD Bioscience, San Jose, CA, USA). A549 cells (1 × 10^5^ cells/ml) were treated with **8** (20 μM) for 30 h, in separate experiments, according to a previously reported method [11]. The DNA contents of the tumor cells were analyzed, and percentages of cells in each phase were calculated.

## 4. Conclusions

Chromatographic separation of the MeOH extract of *Convallaria majalis* (Liliaceae) whole plants yielded 15 steroidal glycosides (**1**–**15**), including nine new compounds (**4**–**6**, **10**–**15**) with a lycotetrose unit. The structures of the new compounds were determined using two-dimensional NMR analyses and chemical methods. The isolated compounds were evaluated for their cytotoxicity against HL-60, A549, HSC-4, and HSC-2 cells. Of these, the spirostanol lycotetroside (**1**) exhibited cytotoxic activity against HL-60, A549, HSC-4, and HSC-2 cells with IC_50_ values ranging from 0.96 to 3.15 μM. Alternatively, the corresponding furostanol lycotetroside (**8**) was cytotoxic to the adherent cell lines of A549, HSC-4, and HSC-2 cells with IC_50_ values of 2.97, 11.04, and 8.25 μM, respectively. Compound **1** caused necrotic cell death in A549 cells in a dose-dependent manner. In contrast, the corresponding furostanol lycotetroside (**8**) induced apoptotic cell death in A549 cells in a time-dependent manner, as was evident by morphological observations and flow cytometry analyses. This study demonstrated that **8** has induced apoptosis against A549 cells through caspase-3/7 and had the potential for anti-lung cancer agent. Further investigation of the pathway of apoptosis induced by **8** should be addressed.

## Figures and Tables

**Table 1:** ^1^H and ^13^C Nuclear magnetic resonance (NMR) spectral data for the sugar moiety of **4** in C_5_D_5_N.

Position			δ_H_		*J* (Hz)	δ_C_	Position			δ_H_		*J* (Hz)	δ_C_
Gal	1′		4.83	d	7.6	102.8	Glc (II)	1′′′		5.59	d	7.5	105.0
	2′		4.40	dd	8.5, 7.6	73.1		2′′′		4.09	dd	8.8, 7.5	76.2
	3′		4.10	m		75.5		3′′′		4.11	dd	8.8, 8.8	77.8
	4′		4.59	br s		79.8		4′′′		4.22	dd	8.8, 8.8	71.7
	5′		3.98	m		75.4		5′′′		3.96	m		78.7
	6′	a	4.68	dd	15.1, 9.4	60.6		6′′′	a	4.60	br d	11.9	62.6
		b	4.21	br d	15.1				b	4.38	br d	11.9	
Glc (I)	1′′		5.19	d	7.5	105.2	Xyl	1′′′′		5.25	d	7.6	104.9
	2′′		4.44	dd	8.8, 7.5	81.3		2′′′′		3.97	dd	8.0, 7.6	75.1
	3′′		4.12	dd	8.8, 8.8	86.7		3′′′′		4.08	dd	8.6, 8.0	78.8
	4′′		3.83	dd	9.5, 8.8	70.5		4′′′′		4.11	m		70.7
	5′′		3.88	m	9.5, 5.6, 2.5	77.6		5′′′′	a	4.24	dd	10.7, 4.9	67.4
	6′′	a	4.53	dd	11.4, 5.6	63.0			b	3.68	dd	10.7, 10.4	
		b	4.02	dd	11.4, 2.5								

**Table 2:** ^13^C NMR spectral data for the aglycone moiety of **4**–**6** and **10**–**15** in C_5_D_5_N.

Position	4	5	6	10	11	12	13	14	15
1	36.3	37.6	37.6	37.1	37.7	37.5	37.4	37.4	36.8
2	30.1	30.1	30.1	30.1	30.1	30.1	30.0	30.1	30.1
3	78.1	78.2	78.0	78.0	78.1	78.1	78.1	78.0	77.8
4	38.9	39.2	39.2	38.7	39.2	39.2	39.2	39.2	39.0
5	166.6	140.5	140.4	141.6	140.5	141.0	141.0	141.2	141.2
6	126.9	122.2	122.1	128.4	122.1	121.6	121.5	121.4	121.1
7	200.6	26.6	26.5	72.6	26.7	31.6	31.9	31.9	31.5
8	49.5	35.5	35.4	40.8	35.0	31.3	31.0	30.9	30.0
9	44.4	43.5	43.5	48.6	43.5	50.2	50.0	50.3	50.4
10	38.5	37.3	37.3	36.9	37.3	37.0	36.9	36.9	37.0
11	20.5	20.3	20.3	21.1	20.5	21.2	20.5	20.6	21.0
12	31.4	31.8	31.8	39.9	31.7	39.6	39.2	38.1	34.9
13	45.9	44.9	44.9	41.2	47.8	43.4	40.3	42.3	46.0
14	84.6	86.3	86.3	56.2	84.7	54.9	56.8	54.0	56.2
15	41.9	39.6	39.6	35.4	42.4	34.4	33.4	35.5	32.2
16	82.2	82.3	82.3	81.6	85.1	84.4	84.2	74.7	144.5
17	58.3	59.2	59.4	63.3	61.5	64.5	67.8	66.6	155.0
18	20.4	19.9	20.0	16.4	17.7	14.1	13.5	13.8	15.7
19	17.1	19.2	19.3	18.8	19.3	19.3	19.3	19.4	19.0
20	42.5	42.4	42.1	40.7	103.9	103.5	76.7	205.5	196.1
21	15.2	14.9	15.1	16.4	11.9	11.8	21.8	30.5	26.9
22	110.0	111.5	111.9	110.6	152.2	152.4	163.0	173.2	
23	26.6	34.3	40.9	37.2	23.7	23.6	91.3	32.2	
24	26.2	72.8	81.5	28.3	31.4	32.3	29.6	29.0	
25	27.5	31.7	38.1	34.4	33.7	33.7	34.8	33.5	
26	65.0	64.2	65.0	75.4	75.2	75.4	75.3	74.7	
27	16.3	9.9	13.5	17.3	17.1	17.1	17.4	16.9	

**Table 3:** Cytotoxic activity of **1**–**15** against HL-60, A549, HSC-4, and HSC-2 cells ^a,b^.

Compound	IC_50_ (µM)			
	HL-60	A549	HSC-4	HSC-2
**1**	2.87 ± 0.15	2.54 ± 0.25	3.15 ± 0.43	0.96 ± 0.01
**8**	>12	2.97 ± 0.06	11.04 ± 0.25	8.25 ± 0.20
doxorubicin	―	―	0.05 ± 0.01	0.11 ± 0.01
etoposide	0.40 ± 0.01	0.72 ± 0.03	―	―
cisplatin	1.33 ± 0.03	2.56 ± 0.05	1.88 ± 0.06	2.17 ± 0.06

^a^ Data are represented as the mean value ± standard error of the mean (SEM) of three experiments performed in triplicate. ^b^ Compounds **2**–**7** and **9**–**15** were inactive against tumor cells (IC_50_ > 12 μM).

**Table 4:** Effects of **8** on cell cycle distribution of A549 cells ^a^.

	% sub G0/G1	% G0/G1	% S	% G2-M
control	4.07 ± 2.00	80.43 ± 5.70	7.47 ± 2.02	6.17 ± 2.30
**8**	14.10 ± 1.33	71.63 ± 4.00	6.67 ± 2.17	5.30 ± 0.38
etoposide	31.15 ± 5.45	25.90 ± 2.40	24.10 ± 6.50	15.40 ± 1.20
cisplatin	40.90 ± 4.24	41.80 ± 4.72	11.90 ± 3.14	4.50 ± 0.61

The cell cycle distribution of A549 cells treated with **8** for 30 h was analyzed using flow cytometry. ^a^ Data are represented as the mean value ± SEM of three experiments performed in triplicate.

## Supplementary Materials

Supplementary materials can be found at http://www.mdpi.com/1422-0067/18/11/2358/s1.
