# Supplementary material for: Steroidal Glycosides from Convallaria majalis Whole Plants and Their Cytotoxic Activity
Source: Int J Mol Sci. 2017 Nov 7;18(11):2358. doi: 10.3390/ijms18112358 (PMC5713327; doi:10.3390/ijms18112358)
Supplement: Supplementary file 1 [file ijms-18-02358-s001.zip › Supplementary materials 1.pdf]

Steroidal glycosides from *Convallaria majalis* whole plants and Their Cytotoxic Activity

Yukiko Matsuo, Daisuke Shinoda, Aina Nakamaru, Kuni Kamohara, Hiroshi Sakagami, and Yoshihiro Mimaki

Supplementary Materials

Table 1. NMR spectral assignments for **1**, **2** and **3**

Table 2. NMR spectral assignments for **7**, **8** and **9**

Table 3. NMR spectral assignments for **4**

Table 4. NMR spectral assignments for **5**

Table 5. NMR spectral assignments for **6**

Table 6. NMR spectral assignments for **10**

Table 7. NMR spectral assignments for **10a**

Table 8. NMR spectral assignments for **11**

Table 9. NMR spectral assignments for **12**

Table 10. NMR spectral assignments for **13**

Table 11. NMR spectral assignments for **13a**

Table 12. NMR spectral assignments for **14**

Table 13. NMR spectral assignments for **15**

Fig. 1-4 Morphological observations by fluorescence microscopy after DAPI staining.

Table 1. ^{13}C-NMR (125 MHz, C_5D_5N) spectral assignments for **1, 2,** and **3**

Position	**1**	**2**	Position	**1**	**2**	Position	**3**	Position	**3**
1	37.4	37.6	Gal 1'	102.7	102.7	1	37.4	Glc 1'	102.5
2	30.1	30.2	2'	73.2	73.2	2	30.0	2'	75.6
3	78.2	78.2	3'	75.6	75.5	3	78.2	3'	76.7
4	39.2	39.9	4'	79.9	79.9	4	39.3	4'	78.3
5	141.0	140.5	5'	75.3	75.3	5	140.9	5'	77.2
6	121.6	122.2	6'	60.5	60.5	6	121.7	6'	62.6
7	32.1	26.5				7	32.2		
8	31.6	35.6	Glc 1''	105.2	105.1	8	31.7	Rha 1''	102.7
9	50.2	43.6	2''	81.3	81.3	9	50.3	2''	72.7
10	37.0	37.4	3''	86.7	86.8	10	37.1	3''	72.9
11	21.0	20.4	4''	70.5	70.5	11	21.1	4''	74.0
12	39.8	31.9	5''	77.6	77.6	12	39.9	5''	70.4
13	40.4	45.1	6''	63.0	63.0	13	40.5	6''	18.6
14	56.5	86.4				14	56.7		
15	32.2	39.2	Glc' 1'''	104.9	104.9	15	32.2		
16	81.1	82.0	2'''	76.2	76.2	16	81.1		
17	62.7	59.7	3'''	77.7	77.7	17	62.9		
18	16.3	20.0	4'''	71.0	71.0	18	16.4		
19	19.4	19.3	5'''	78.7	78.7	19	19.4		
20	42.4	42.5	6'''	62.7	62.4	20	42.0		
21	14.9	15.2				21	15.0		
22	109.7	110.0	Xyl 1''''	104.8	104.8	22	109.3		
23	26.3	26.6	2''''	75.1	75.1	23	31.8		
24	26.1	26.2	3''''	78.7	78.6	24	29.3		
25	27.5	27.6	4''''	70.7	70.7	25	30.6		
26	65.0	65.1	5''''	67.3	67.3	26	66.9		
27	16.3	16.3				27	17.3		

Table 2. ^{13}C-NMR (125 MHz, C_5D_5N) spectral assignments for **7**, **8**, and **9**.

Position	**7**	Position		Position	**8**	**9**	Position	**8**	**9**
1	37.7	Glc 1'	105.1	1	37.5	37.7	Gal 1'	102.7	102.6
2	32.6	2'	75.2	2	30.0	30.1	2'	73.1	73.1
3	71.3	3'	78.6	3	78.2	78.2	3'	75.5	75.5
4	43.4	4'	71.7	4	39.2	39.2	4'	79.8	79.8
5	142.0	5'	78.5	5	141.0	140.5	5'	75.2	75.2
6	121.1	6'	62.8	6	121.6	122.2	6'	60.5	60.5
7	32.3			7	32.3	26.7			
8	31.7			8	31.6	35.0	Glc 1''	105.1	105.1
9	50.4			9	50.3	43.6	2''	81.3	81.3
10	37.0			10	37.1	37.3	3''	86.7	86.8
11	21.2			11	21.1	20.4	4''	70.5	70.4
12	40.0			12	39.9	32.0	5''	77.6	77.6
13	40.8			13	40.8	45.4	6''	62.8	62.9
14	56.7			14	56.6	86.3			
15	32.5			15	32.4	40.0	Glc' 1'''	104.8	104.8
16	81.1			16	81.3	81.7	2'''	76.2	76.2
17	63.8			17	63.8	60.5	3'''	77.7	77.7
18	16.5			18	16.4	20.1	4'''	71.0	71.0
19	19.5			19	19.4	19.3	5'''	78.6	78.5
20	40.6			20	40.7	40.8	6'''	62.5	62.4
21	16.4			21	16.4	16.7			
22	110.7			22	110.7	113.0	Xyl 1''''	104.9	104.9
23	37.1			23	37.0	37.1	2''''	75.1	75.2
24	28.3			24	28.3	28.3	3''''	78.7	78.7
25	34.4			25	34.4	34.4	4''''	70.7	70.7
26	75.3			26	75.4	75.4	5''''	67.3	67.3
27	17.4			27	17.4	17.4			
							Glc'' 1'''''	105.1	105.1
							2'''''	75.2	75.2
							3'''''	78.5	78.4
							4'''''	71.7	71.7
							5'''''	78.4	78.4
							6'''''	62.8	62.8

Table 3. ^{1}H- and ^{13}C-NMR (500 and 125 MHz, C_5D_5N) spectral assignments for **4**

Position	δ_H		J (Hz)	δ_C	Position		δ_H		J (Hz)	δ_C
1 ax	0.98	m		36.3	Gal 1'		4.83	d	7.6	102.8
eq	1.69	br d	12.9		2'		4.40	dd	8.5, 7.6	73.1
2 ax	1.70	m		30.1	3'		4.10	m		75.5
eq	2.09	br dd	15.5, 5.5		4'		4.59	br s		79.8
3	3.86	m	$W_{1/2}$ = 27.4	78.1	5'		3.98	m		75.4
4 ax	2.44	t	12.2	38.9	6'	a	4.68	dd	15.1, 9.4	60.6
eq	2.71	br d	12.2			b	4.21	br d	15.1	
5	-			166.6						
6	5.75	s		126.9	Glc 1"		5.19	d	7.5	105.2
7	-			200.6	2"		4.44	dd	8.8, 7.5	81.3
8	2.74	d	13.1	49.5	3"		4.12	dd	8.8, 8.8	86.7
9	2.41	m		44.4	4"		3.83	dd	9.5, 8.8	70.5
10	-			38.5	5"		3.88	m	9.5, 5.6, 2.5	77.6
11 (2H)	1.54	m		20.5	6"	a	4.53	dd	11.4, 5.6	63.0
12 ax	1.41	m		31.4		b	4.02	dd	11.4, 2.5	
eq	2.21	m								
13	-			45.9	Glc' 1'''		5.59	d	7.5	105.0
14	-			84.6	2'''		4.09	dd	8.8, 7.5	76.2
15 a	3.48	m		41.9	3'''		4.11	dd	8.8, 8.8	77.8
b	2.21	m			4'''		4.22	dd	8.8, 8.8	71.7
16	5.06	dd	12.7, 9.0	82.2	5'''		3.96	m		78.7
17	2.72	t	8.3	58.3	6'''	a	4.60	br d	11.9	62.6
18	1.04	s		20.4		b	4.38	br d	11.9	
19	0.99	s		17.1						
20	2.02	t	6.7	42.5	Xyl 1''''		5.25	d	7.6	104.9
21	1.21	d	7.0	15.2	2''''		3.97	dd	8.0, 7.6	75.1
22	-			110.0	3''''		4.08	dd	8.6, 8.0	78.8
23 ax	1.93	ddd	13.7, 13.7, 4.7	26.6	4''''		4.11	m		70.7
eq	1.47	br dd	13.7, 3.6		5''''	a	4.24	dd	10.7, 4.9	67.4
24 ax	2.12	br dd	13.7, 12.3	26.2		b	3.68	dd	10.7, 10.4	
eq	1.34	br d	12.3							
25	1.55	m		27.5						
26 ax	4.02	dd	10.9, 2.5	65.0						
eq	3.31	br d	10.9							
27	1.07	d	7.0	16.3						

Table 4. ^{1}H- and ^{13}C-NMR (500 and 125 MHz, C_5D_5N) spectral assignments for **5**

Position	δ_H		J (Hz)	δ_C	Position	δ_H		J (Hz)	δ_C
1 ax	1.01	m		37.6	Gal 1'	4.86	d	7.5	102.7
eq	1.72	br d	11.6		2'	4.41	dd	8.5, 7.5	73.2
2 ax	1.69	t	12.9	30.1	3'	4.08	m		75.5
eq	2.06	m			4'	4.58	br s		79.8
3	3.88	m	$W_{1/2}$ = 23.3	78.2	5'	3.96	m		75.3
4 ax	2.44	dd	11.5, 10.5	39.2	6' a	4.66	dd	13.5, 9.4	60.5
eq	2.65	dd	11.5, 3.2		b	4.16	m		
5	-			140.5					
6	5.37	br s		122.2	Glc 1''	5.17	d	7.9	105.1
7 ax	2.48	m		26.6	2''	4.40	dd	8.5, 7.9	81.3
eq	1.84	br d	6.6		3''	4.16	dd	8.8, 8.5	86.7
8	2.02	m		35.5	4''	3.80	dd	9.4, 8.8	70.4
9	1.78	m		43.5	5''	3.87	m		77.6
10	-			37.3	6'' a	4.50	br d	11.8	62.9
11 (2H)	1.51	m		20.3	b	4.03	br d	11.8	
12 ax	1.41	br d	12.1	31.8					
eq	2.24	m			Glc' 1'''	5.57	d	7.5	104.8
13	-			44.9	2'''	4.06	dd	8.8, 7.5	76.2
14	-			86.3	3'''	4.08	dd	9.0, 8.8	77.7
15 a	2.28	br d	7.6	39.6	4'''	4.22	dd	9.0, 9.0	71.0
b	1.78	m			5'''	3.96	m		78.6
16	5.21	m		82.3	6''' a	4.57	br d	10.8	62.4
17	2.73	t	7.3	59.2	b	4.38	br d	10.8	
18	1.00	s		19.9					
19	0.96	s		19.2	Xyl 1''''	5.23	d	7.8	104.9
20	2.07	m		42.4	2''''	3.96	dd	8.2, 7.8	75.0
21	1.14	d	6.8	14.9	3''''	4.09	dd	9.0, 8.2	78.7
22	-			111.5	4''''	4.13	m		70.7
23 (2H)	2.15	m		34.3	5'''' a	4.23	br d	10.6	67.3
24	4.83	ddd	10.9, 5.5, 5.5	72.8	b	3.67	dd	10.6, 10.4	
25	2.24	m		31.7					
26 ax	3.89	br d	10.6	64.2	Glc'' 1'''''	5.04	d	7.7	101.2
eq	3.47	br d	10.6		2'''''	4.07	dd	8.8, 7.7	75.3
27	1.31	d	6.9	9.9	3'''''	4.26	dd	9.0, 8.8	78.7
					4'''''	4.30	dd	9.0, 9.0	71.6
					5'''''	3.94	m		78.4
					6''''' a	4.51	br d	10.8	62.6
					b	4.39	br d	10.8	

Table5 . ^{1}H- and ^{13}C-NMR (500 and 125 MHz, C_5D_5N) spectral assignments for **6**

Position	δ_H		*J* (Hz)	δ_C	Position		δ_H		*J* (Hz)	δ_C
1 ax	1.01	m		37.6	Gal 1'		4.87	d	7.6	102.7
eq	1.71	br dd	12.9, 6.4		2'		4.42	dd	8.5, 7.6	73.1
2 ax	1.70	t	7.9	30.1	3'		4.08	dd	8.5, 8.5	75.5
eq	2.06	br dd	10.1, 6.8		4'		4.58	br s		79.8
3	3.88	m	$W_{1/2}$ = 22.5	78.0	5'		3.96	m		75.3
4 ax	2.44	t	10.8	39.2	6'	a	4.65	m		60.5
eq	2.64	dd	13.9, 3.5			b	4.16	m		
5	-			140.4						
6	5.37	d	4.2	122.1	Glc 1"		5.17	d	7.9	105.1
7 ax	2.46	m		26.5	2"		4.41	dd	8.5, 7.9	81.3
eq	1.81	m			3"		4.16	dd	8.9, 8.5	86.8
8	1.97	m		35.4	4"		3.81	dd	9.1, 8.9	70.4
9	1.78	m		43.5	5"		3.88	m		77.6
10	-			37.3	6"	a	4.54	br d	11.1	62.9
11 (2H)	1.52	m		20.3		b	4.04	br d	11.1	
12 ax	1.39	br d	9.7	31.8						
eq	2.22	m			Glc' 1'''		5.57	d	7.5	104.8
13	-			44.9	2'''		3.97	dd	8.8, 7.5	76.2
14	-			86.3	3'''		4.08	dd	9.2, 8.8	77.7
15 a	2.28	dd	10.6, 7.5	39.6	4'''		4.21	dd	9.2, 9.2	71.0
b	1.82	m			5'''		4.00	m		78.6
16	5.05	m		82.3	6'''	a	4.57	br d	10.8	62.4
17	2.73	t	7.6	59.4		b	4.37	bb	10.8, 5.4	
18	0.99	s		20.0						
19	0.95	s		19.3	Xyl 1''''		5.23	d	7.8	104.9
20	2.07	m		42.1	2''''		3.95	dd	8.3, 7.8	75.0
21	1.01	d	6.9	15.1	3''''		4.07	dd	8.5, 8.3	78.7
22	-			111.9	4''''		4.12	m		70.7
23 ax	2.00	dd	12.4, 12.4	40.9	5''''	a	4.22	dd	10.7, 4.6	67.3
eq	2.73	dd	12.4, 5.3			b	3.67	dd	10.7, 10.5	
24	4.06	m		81.5						
25	1.91	m		38.1	Glc" 1'''''		4.94	d	7.7	106.3
26 ax	3.58	dd	11.8, 11.8	65.0	2'''''		4.06	dd	8.6, 7.7	75.7
eq	3.60	dd	11.8, 4.0		3'''''		4.21	dd	8.8, 8.6	78.6
27	1.13	d	6.5	13.5	4'''''		4.28	dd	8.8, 8.8	71.7
					5'''''		3.90	m		78.2
					6'''''	a	4.52	br d	13.7	62.8
						b	4.39	br d	13.7	

Table 6. ^{1}H- and ^{13}C-NMR (500 and 125 MHz, C_5D_5N) spectral assignments for **10**

Position	δ_H		J (Hz)	δ_C	Position		δ_H		J (Hz)	δ_C
1 ax	0.96	m		37.1	Gal 1'		4.87	d	7.6	102.8
eq	1.66	m			2'		4.41	dd	8.5, 7.6	73.1
2 ax	1.69	m		30.1	3'		4.09	m		75.5
eq	2.10	m			4'		4.58	br s		79.8
3	3.92	m	$W_{1/2}$ = 25.6	78.0	5'		3.96	m		75.3
4 ax	2.41	dd	13.6, 11.4	38.7	6'	a	4.67	dd	14.5, 9.4	60.5
eq	2.69	dd	13.6, 2.4			b	4.17	br d	14.5	
5	-			141.6						
6	5.60	br s		128.4	Glc 1''		5.17	d	7.9	105.1
7	4.00	m		72.6	2''		4.41	dd	8.5, 7.9	81.3
8	1.79	m		40.8	3''		4.16	dd	8.8, 8.5	86.7
9	1.06	m		48.6	4''		3.81	dd	9.2, 8.8	70.4
10	-			36.9	5''		3.87	m		77.6
11 (2H)	1.44	m		21.1	6''	a	4.53	m		62.9
12 ax	1.74	br d	13.5	39.9		b	4.05	m		
eq	1.14	m								
13	-			41.2	Glc' 1'''		5.56	d	7.5	104.8
14	1.35	m		56.2	2'''		4.08	dd	8.8, 7.5	76.2
15 a	2.85	m		35.4	3'''		4.10	dd	9.0, 8.8	77.7
b	2.04	m			4'''		4.21	dd	9.0, 9.0	71.0
16	5.04	m		81.6	5'''		3.93	m		78.6
17	1.94	m		63.3	6'''	a	4.58	br d	12.7	62.4
18	0.94	s		16.4		b	4.38	dd	12.7, 5.4	
19	0.85	s		18.8						
20	2.24	t	6.6	40.7	Xyl 1''''		5.22	d	7.8	104.9
21	1.34	d	6.9	16.4	2''''		3.97	dd	8.3, 7.8	75.0
22	-			110.6	3''''		4.09	dd	8.9, 8.3	78.7
23 a	2.08	m		37.2	4''''		4.11	m		70.7
b	1.96	m			5''''	a	4.22	m		67.3
24 a	2.09	m		28.3		b	3.66	dd	10.7, 10.3	
b	1.68	m								
25	1.92	m		34.4	Glc'' 1'''''		4.80	d	7.7	105.1
26 a	3.96	dd	9.2, 5.6	75.4	2'''''		4.02	dd	8.8, 7.7	75.2
b	3.45	dd	9.2, 7.0		3'''''		4.24	dd	8.8, 8.8	78.6
27	1.01	d	6.7	17.3	4'''''		4.23	dd	8.8, 8.8	71.7
					5'''''		3.92	m		78.4
					6'''''	a	4.55	br d	12.7	62.8
7-OH	5.71	br d	7.9			b	4.38	dd	12.7, 5.4	

Table 7. ^{1}H- and ^{13}C-NMR (500 and 125 MHz, C_5D_5N) spectral assignments for **10a**

Position	δ_H	J (Hz)	δ_C	Position	δ_H	J (Hz)	δ_C
1 ax	0.95 ddd	14.4, 13.3, 3.3	37.2	Gal 1'	4.89 d	7.6	102.8
eq	1.66 br dd	14.4, 3.3		2'	4.43 dd	8.2, 7.6	73.1
2 ax	1.67 m		30.1	3'	4.08 dd	8.6, 8.2	75.5
eq	2.11 m			4'	4.58 br s		79.8
3	3.91 m	$W_{1/2}$ = 28.7	78.0	5'	3.97 m		75.3
4 ax	2.42 dd	13.5, 11.7	38.8	6' a	4.67 dd	14.8, 9.6	60.5
eq	2.69 dd	13.5, 2.4		b	4.18 m		
5	-		141.6				
6	5.61 br s		128.5	Glc 1''	5.18 d	7.9	105.1
7	4.01 m		72.6	2''	4.43 dd	8.7, 7.9	81.3
8	1.78 ddd	15.3, 13.1, 8.7	40.7	3''	4.16 dd	9.0, 8.7	86.7
9	1.05 ddd	8.7, 6.8, 3.0	48.6	4''	3.82 dd	9.0, 9.0	70.4
10	-		37.0	5''	3.88 ddd	9.0, 5.7, 2.1	77.6
11 ax	1.37 m		21.2	6'' a	4.52 dd	11.0, 5.7	63.0
eq	1.45 m			b	4.06 dd	11.0, 2.1	
12 ax	1.68 ddd	12.0, 12.0, 4.3	39.9				
eq	1.11 br d	12.0		Glc' 1'''	5.58 d	7.5	104.9
13	-		40.9	2'''	4.06 dd	8.8, 7.5	76.2
14	1.32 m		56.3	3'''	4.11 dd	8.8, 8.8	77.7
15 a	2.84 ddd	13.2, 6.5, 6.5	35.1	4'''	4.22 dd	8.8, 8.8	71.0
b	2.00 ddd	13.2, 13.2, 6.5		5'''	3.94 m		78.6
16	4.62 m		81.7	6''' a	4.59 br d	12.6	62.5
17	1.81 dd	8.3, 6.5	62.3	b	4.38 dd	12.6, 3.7	
18	0.88 s		16.4				
19	0.84 s		18.9	Xyl 1''''	5.23 d	7.8	104.8
20	1.92 m		42.5	2''''	3.97 dd	8.1, 7.8	75.1
21	1.17 d	7.0	14.9	3''''	4.09 dd	8.6, 8.1	78.7
22	-		109.7	4''''	4.13 m		70.7
23 ax	1.90 ddd	13.7, 13.7, 4.7	26.4	5'''' a	4.23 dd	11.4, 4.9	67.3
eq	1.43 br dd	13.7, 2.1		b	3.68 dd	11.4, 10.7	
24 ax	2.14 m		26.2				
eq	1.35 m						
25	1.57 m		27.5				
26 ax	4.05 dd	11.0, 3.0	65.0				
eq	3.35 br d	11.0					
27	1.07 d	7.1	16.3				
7-OH	5.73 br d	7.8					

Table 8. ^{1}H- and ^{13}C-NMR (500 and 125 MHz, C_5D_5N) spectral assignments for **11**

Position	δ_H		J (Hz)	δ_C	Position			δ_H		J (Hz)	δ_C
1 ax	1.02	m		37.7	Gal	1'		4.86	d	7.6	102.6
eq	1.73	br d	12.8			2'		4.40	dd	8.5, 7.6	73.1
2 ax	1.72	t	12.8	30.1		3'		4.11	m		75.5
eq	2.08	br d	10.4			4'		4.58	br s		79.8
3	3.85	m	$W_{1/2}$ = 23.8	78.1		5'		3.97	m		75.2
4 ax	2.44	t	12.5	39.2		6'	a	4.65	dd	12.1, 8.1	60.5
eq	2.66	dd	13.5, 2.0				b	4.16	br d	12.1	
5	-			140.5							
6	5.38	br d	5.6	122.1	Glc	1''		5.17	d	7.9	105.1
7	2.45	m		26.7		2''		4.41	dd	8.8, 7.9	81.3
	1.86	m				3''		4.14	dd	8.8, 8.8	86.7
8	1.96	m		35.0		4''		3.80	dd	9.3, 8.8	70.4
9	1.79	m		43.5		5''		3.85	m		77.5
10	-			37.3		6''	a	4.51	br d	10.8	62.9
11 (2H)	1.54	m		20.5			b	4.04	dd	10.8, 5.3	
12 ax	1.47	m		31.7							
eq	2.31	br d	13.5		Glc'	1'''		5.56	d	7.5	104.8
13	-			47.8		2'''		4.05	dd	8.8, 7.5	76.2
14	-			84.7		3'''		4.07	dd	9.0, 8.8	77.7
15 a	2.42	dd	13.0, 7.7	42.4		4'''		4.19	dd	9.0, 9.0	71.0
b	1.92	dd	13.0, 5.4			5'''		4.07	m		78.5
16	5.27	m		85.1		6'''	a	4.54	br d	12.4	62.3
17	3.36	d	9.7	61.5			b	4.35	dd	12.4, 5.4	
18	0.93	s		17.7							
19	0.98	s		19.3	Xyl	1''''		5.22	d	7.8	104.9
20	-			103.9		2''''		3.95	dd	8.4, 7.8	75.2
21	1.67	s		11.9		3''''		4.08	dd	9.2, 8.4	78.7
22	-			152.2		4''''		4.10	m		70.7
23 (2H)	2.24	m		23.7		5''''	a	4.22	m		67.3
24 a	1.88	m		31.4			b	3.67	dd	10.6, 10.4	
b	1.48	m									
25	1.96	m		33.7	Glc''	1'''''		4.83	d	7.8	105.1
26 a	3.94	dd	9.3, 6.6	75.2		2'''''		4.03	dd	8.4, 7.8	75.2
b	3.50	dd	9.3, 7.3			3'''''		4.25	dd	8.8, 8.4	78.5
27	1.04	d	6.6	17.1		4'''''		4.23	dd	8.8, 8.4	71.7
						5'''''		3.95	m		78.4
						6'''''	a	4.56	br d	11.5	62.8
							b	4.38	br d	11.5	

Table 9. ^{1}H- and ^{13}C-NMR (500 and 125 MHz, C_5D_5N) spectral assignments for **12**

positions		δ_H		J (Hz)	δ_C		positions			δ_H		J (Hz)	δ_C
1	ax	0.96	m		37.5		Gal	1'		4.88	d	7.5	102.7
	eq	1.67	br d	12.6				2'		4.39	dd	8.3, 7.5	73.1
2	ax	1.70	br d	11.7	30.1			3'		4.11	m		75.5
	eq	2.09	m					4'		4.59	br s		79.8
3		3.87		$W_{1/2}$ = 23.6	78.1			5'		3.97	m		75.3
4	ax	2.42	t	12.3	39.2			6'	a	4.66	dd	14.2, 9.1	60.5
	eq	2.65	dd	10.6, 2.4					b	4.16	br d	9.1	
5		-			141.0								
6		5.30	br d	3.9	121.6		Glc(Ⅰ)	1"		5.18	d	7.8	105.1
7	ax	1.46	m		31.6			2"		4.41	dd	8.8, 7.8	81.3
	eq	2.09	m					3"		4.16	dd	9.2, 8.8	86.7
8		1.48	m		31.3			4"		3.81	dd	9.2, 8.9	70.4
9		0.87	m		50.2			5"		3.87	m		77.5
10		-			37.0			6"	a	4.51	br d	9.7	62.9
11	(2H)	1.42	m		21.2				b	4.05	br d	9.7	
12	ax	1.13	m		39.6								
	eq	1.73	br d	12.5			Glc(Ⅱ)	1"'		5.56	d	7.3	104.8
13		-			43.4			2"'		4.07	dd	8.8, 7.3	76.2
14		0.83	m		54.9			3"'		4.09	dd	9.2, 8.8	77.7
15	a	1.47	br d	7.1	34.4			4"'		4.20	m		71.0
	b	2.09	br d	7.0				5"'		3.91	m		78.4
16		4.80	m		84.4			6"'	a	4.57	br d	9.0	62.4
17		2.44	d	9.9	64.5				b	4.37	br d	9.0	
18		0.70	s		14.1								
19		0.89	s		19.3		Xyl	1""		5.22	d	7.7	104.9
20		-			103.5			2""		3.98	dd	8.5, 7.7	75.0
21		1.62	s		11.8			3""		4.08	m		78.7
22		-			152.4			4""		4.09	m		70.7
23	a	2.23	m		23.6			5""	a	4.22	m		67.3
	b	2.17	m						b	3.67	dd	10.7, 10.6	
24	a	1.87	m		32.3								
	b	1.50	m				Glc(Ⅲ)	1""'		4.82	d	7.7	105.1
25		1.94	m		33.7			2""'		4.01	br d	7.5	75.2
26	a	3.48	dd	9.1, 7.1	75.4			3""'		4.23	dd	8.5, 8.0	78.5
	b	4.05	m					4""'		4.22	dd	8.5, 8.0	71.7
27		1.03	d	6.6	17.1			5""'		3.96	m		78.6
								6""'		4.55	br d	8.9	62.8
										4.38	br d	8.9	

Table 10. ^{1}H- and ^{13}C-NMR (500 and 125 MHz, C_5D_5N) spectral assignments for **13**

Position	δ_H		J (Hz)	δ_C	Position		δ_H		J (Hz)	δ_C
1 ax	0.96	ddd	12.8, 11.0, 3.0	37.4	Gal 1'		4.89	d	7.6	102.7
eq	1.66	br d	11.1		2'		4.42	dd	8.6, 7.6	73.1
2 ax	1.68	t	13.6	30.0	3'		4.11	m		75.5
eq	2.11	m			4'		4.60	br d	2.3	79.8
3	3.89	m	$W_{1/2}$ = 22.8	78.1	5'		3.97	m		75.3
4 ax	2.42	dd	13.0, 11.8	39.2	6'	a	4.68	dd	15.8, 9.6	60.5
eq	2.65	dd	13.0, 2.3			b	4.18	m		
5	-			141.0						
6	5.29	br s		121.5	Glc 1''		5.19	d	7.9	105.1
7 ax	1.49	m		31.9	2''		4.42	dd	8.6, 7.9	81.3
eq	1.83	dd	13.5, 2.3		3''		4.16	dd	8.8, 8.6	86.7
8	1.50	m		31.0	4''		3.82	dd	9.2, 8.8	70.4
9	0.84	m		50.0	5''		3.88	m		77.6
10	-			36.9	6''	a	4.53	br d	11.6	62.9
11 (2H)	1.39	m		20.5		b	4.05	br d	11.6	
12 ax	1.17	br d	12.1	39.2						
eq	1.89	m			Glc' 1'''		5.58	d	7.4	104.8
13	-			40.3	2'''		4.08	dd	8.8, 7.4	76.2
14	0.96	m		56.8	3'''		4.10	dd	9.0, 8.8	77.7
15 a	2.05	m		33.4	4'''		4.23	dd	9.0, 9.0	71.0
b	1.48	m			5'''		3.93	m		78.6
16	5.20	m		84.2	6'''	a	4.59	br d	12.4	62.4
17	2.23	d	6.3	67.8		b	4.38	br d	12.4	
18	0.90	s		13.5						
19	0.90	s		19.3	Xyl 1''''		5.24	d	7.8	104.9
20	-			76.7	2''''		3.98	dd	8.5, 7.8	75.1
21	1.73	s		21.8	3''''		4.09	dd	9.2, 8.5	78.7
22	-			163.0	4''''		4.12	m		70.7
23	4.53	br d	13.8	91.3	5''''	a	4.23	dd	10.6, 4.7	67.3
24 a	2.52	m		29.6		b	3.68	dd	10.6, 10.4	
b	2.15	m								
25	2.09	m		34.8	Glc'' 1'''''		4.85	d	7.7	105.1
26 a	4.16	dd	9.1, 6.0	75.3	2'''''		4.04	dd	8.8, 7.7	75.3
b	3.53	dd	9.1, 7.1		3'''''		4.25	dd	9.0, 8.8	78.7
27	1.08	d	6.3	17.4	4'''''		4.23	dd	9.0, 9.0	71.6
					5'''''		3.95	m		78.4
					6'''''	a	4.55	m		62.7
						b	4.41	br d	11.9	

Table 11. ^{1}H- and ^{13}C-NMR (500 and 125 MHz, C_5D_5N) spectral assignments for **13a**

Position	δ_H		J (Hz)	δ_C	Position		δ_H		J (Hz)	δ_C
1 ax	0.96	ddd	12.0, 12.0, 3.9	37.4	Gal 1'		4.89	d	7.7	102.8
eq	1.67	br d	12.0		2'		4.42	dd	8.8, 7.7	73.2
2 ax	1.71	m		30.1	3'		4.10	m		75.6
eq	2.09	m			4'		4.60	br d	2.6	79.9
3	3.89	m	$W_{1/2}$ = 23.6	78.2	5'		3.98	m		75.3
4 ax	2.43	br d	13.3	39.2	6'	a	4.68	m		60.5
eq	2.66	dd	13.3, 2.5			b	4.18	br d	13.2	
5	-			141.1						
6	5.32	br d	5.0	121.7	Glc 1"		5.19	d	7.9	105.2
7 ax	1.48	br dd	13.3, 13.3	32.3	2"		4.44	dd	8.6, 7.9	81.3
eq	1.87	br d	13.3		3"		4.18	dd	8.8, 8.6	86.7
8	1.59	m		30.8	4"		3.83	dd	9.2, 8.8	70.5
9	0.85	m		50.3	5"		3.88	m		77.6
10	-			37.1	6"	a	4.53	dd	11.0, 2.4	63.0
11 (2H)	1.42	m		20.9		b	4.06	dd	11.0, 4.6	
12 ax	1.21	m		41.4						
eq	1.99	br d	12.1		Glc' 1"'		5.58	d	7.4	104.9
13	-			41.6	2"'		4.09	dd	8.8, 7.4	76.2
14	0.88	m		56.3	3"'		4.11	dd	9.0, 8.8	77.7
15 a	2.04	m		34.2	4"'		4.21	dd	9.0, 9.0	71.0
b	1.48	m			5"'		3.93	m		78.7
16	5.06	m		82.8	6"'	a	4.59	br d	12.4	62.5
17	2.44	d	8.5	70.2		b	4.38	dd	12.4, 4.7	
18	1.19	s		15.8						
19	0.93	s		19.4	Xyl 1""		5.24	d	7.8	105.0
20	-			82.6	2""		3.98	dd	8.3, 7.8	75.1
21	1.73	s		20.5	3""		4.10	dd	9.0, 8.3	78.7
22	-			110.1	4""		4.12	m		70.7
23 ax	1.79	br dd	12.9, 5.8	30.2	5""	a	4.23	dd	10.9, 5.1	67.4
eq	2.37	br d	12.9			b	3.69	dd	10.9, 9.8	
24 (2H)	1.70	m		29.5						
25	1.69	m		30.6						
26 ax	3.70	dd	11.5, 10.0	67.3						
eq	3.60	dd	11.5, 3.8							
27	0.75	d	5.5	17.3						

Table 12. ^{1}H- and ^{13}C-NMR (500 and 125 MHz, C_5D_5N) spectral assignments for **14**

Position	δ_H		*J* (Hz)	δ_C	Position			δ_H		*J* (Hz)	δ_C
1 ax	0.94	m		37.4	Gal	1'		4.89	d	7.6	102.7
eq	1.64	br d	13.4			2'		4.42	dd	8.2, 7.6	73.2
2 ax	1.68	t	15.3	30.1		3'		4.07	m		75.5
eq	2.08	m				4'		4.60	br s		79.8
3	3.89	m	$W_{1/2}$ = 20.3	78.0		5'		3.97	m		75.3
4 ax	2.42	dd	14.2, 13.4	39.2		6'	a	4.68	dd	14.5, 9.4	60.5
eq	2.65	dd	13.4, 2.1				b	4.18	m		
5	-			141.2							
6	5.30	br d	4.3	121.4	Glc	1''		5.19	d	7.9	105.2
7 ax	1.84	m		31.9		2''		4.43	dd	8.8, 7.9	81.3
eq	1.48	m				3''		4.17	dd	8.8, 8.8	86.7
8	1.48	m	13.1	30.9		4''		3.83	dd	9.0, 8.8	70.5
9	0.86	m		50.3		5''		3.87	br dd	9.0, 1.7	77.6
10	-			36.9		6''	a	4.53	br d	11.0	63.0
11 (2H)	1.42	m		20.6			b	4.05	dd	11.0, 1.7	
12 ax	1.08	br dd	16.5, 10.2	38.1							
eq	2.16	m			Glc'	1'''		5.58	d	7.4	104.9
13	-			42.3		2'''		4.09	dd	8.8, 7.4	76.2
14	0.79	m		54.0		3'''		4.11	dd	8.8, 8.8	77.7
15 a	2.38	m		35.5		4'''		4.22	dd	9.0, 8.8	71.0
b	1.27	ddd	13.0, 13.0, 4.3			5'''		3.94	m		78.6
16	5.66	m		74.7		6'''	a	4.59	br d	13.1	62.5
17	2.48	d	7.6	66.6			b	4.39	br d	13.1	
18	1.20	s		13.8							
19	0.87	s		19.4	Xyl	1''''		5.24	d	7.8	104.9
20	-			205.5		2''''		3.98	dd	8.0, 7.8	75.1
21	2.13	s		30.5		3''''		4.08	dd	8.8, 8.0	78.7
22	-			173.2		4''''		4.10	m		70.7
23 a	1.84	m		32.2		5''''	a	4.23	dd	10.9, 4.4	67.3
b	1.47	m					b	3.68	dd	10.9, 10.4	
24 a	1.96	m		29.0							
b	1.59	m			Glc''	1'''''		4.80	d	7.8	105.2
25	1.88	m		33.5		2'''''		4.03	dd	9.0, 7.8	75.2
26 a	3.97	dd	9.5, 6.1	74.7		3'''''		4.23	dd	9.0, 9.0	78.6
b	3.45	dd	9.5, 6.4			4'''''		0.22	dd	9.0, 9.0	71.5
27	0.92	d	8.3	16.9		5'''''		3.92	m		78.5
						6'''''	a	4.58	br d	13.1	62.8
							b	4.38	br d	13.1	

Table 13. ^{1}H- and ^{13}C-NMR (600 and 150 MHz, C_5D_5N) spectral assignments for **15**

Position	δ_H		*J* (Hz)	δ_C	Position			δ_H		*J* (Hz)	δ_C
1 ax	0.88	br dd	12.4, 12.4	36.8	Gal	1'		4.88	d	7.8	102.5
eq	1.56	m				2'		4.43	dd	8.4, 7.8	72.9
2 ax	1.65	m		30.1		3'		4.09	m		75.3
eq	2.05	br d	15.5, 5.5			4'		4.58	br d	3.6	79.6
3	3.87	m	$W_{1/2}$ = 18.9	77.8		5'		3.95	m		75.1
4 ax	2.39	dd	13.2, 10.8	39.0		6'	a	4.66	dd	15.0, 9.6	60.3
eq	2.64	dd	13.2, 2.4				b	4.15	m		
5	-			141.2							
6	5.29	br d	5.4	121.1	Glc	1"		5.18	d	7.8	105.0
7 ax	1.82	m		31.5		2"		4.43	dd	8.4, 7.8	81.1
eq	1.51	m				3"		4.16	dd	9.0, 8.4	86.4
8	1.49	m		30.0		4"		3.81	dd	9.0, 9.0	70.2
9	0.87	m		50.4		5"		3.87	ddd	9.0, 5.4, 2.4	77.4
10	-			37.0		6"	a	4.50	dd	9.6, 2.4	62.7
11 (2H)	1.44	m		21.0			b	4.03	dd	9.6, 5.4	
12 ax	1.32	m		34.9							
eq	2.58	ddd	12.0, 3.6, 3.0		Glc'	1'''		5.58	d	7.8	104.7
13	-			46.0		2'''		4.05	dd	8.8, 7.8	76.0
14	1.30	m		56.2		3'''		4.09	dd	9.0, 8.8	77.8
15 a	2.11	ddd	16.8, 6.0, 2.8	32.2		4'''		4.20	dd	9.6 ,9.0	70.7
b	1.84	br dd	16.8, 2.8			5'''		3.92	m	9.6, 4.2, 1.8	78.4
16	6.57	br s		144.5		6'''	a	4.58	dd	11.4, 1.8	62.2
17	-			155.0			b	4.36	dd	11.4, 4.2	
18	0.90	s		15.7							
19	0.86	s		19.0	Xyl	1''''		5.23	d	7.8	104.8
20	-			196.1		2''''		3.95	dd	8.4, 7.8	74.8
21	2.22	s		26.9		3''''		4.05	dd	8.8, 8.4	78.5
						4''''		4.09	m		70.5
						5''''	a	4.21	dd	11.4, 4.8	67.1
							b	3.66	dd	11.4, 10.8	

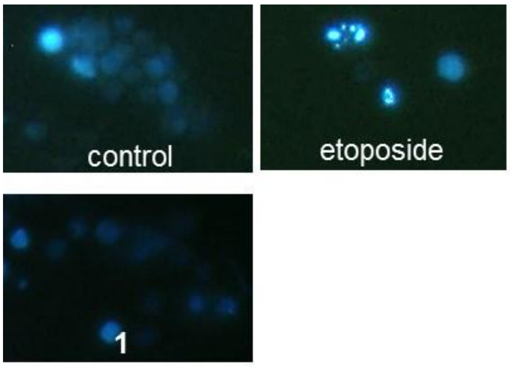

Fig. 1 Morphological observations of representative fields of HL-60 cells stained with DAPI to evaluate fragmented and condensed unclear chromatins after treatment with **1** (20 nM) or etoposide (15 nM) for 3h.

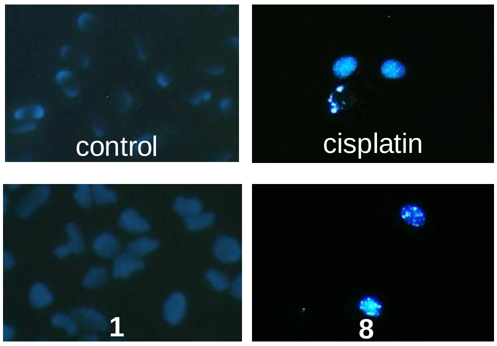

Fig. 2. Morphological observations of representative fields of A549 cells stained with DAPI to evaluate fragmented and condensed unclear chromatins after treatment with **1** (20 nM) for 3h, **8** (20 nM) or cisplatin (33 nM) for 24 h.

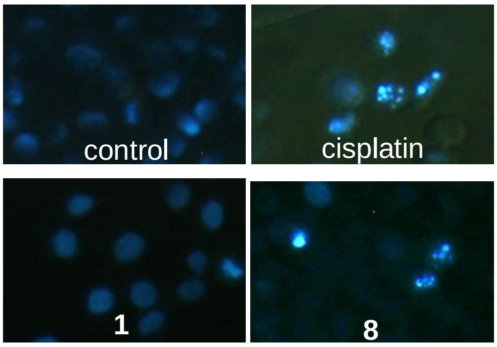

Fig.3. Morphological observations of representative fields of HSC-4 cells stained with DAPI to evaluate fragmented and condensed unclear chromatins after treatment with **1** (10 nM) for 3h, **8** (20 nM) or cisplatin (33 nM) for 48h.

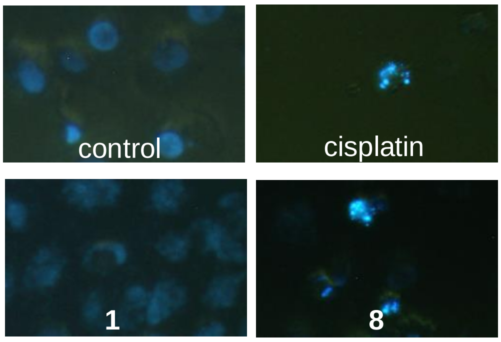

Fig. 4. Morphological observations of representative fields of HSC-2 cells stained with DAPI to evaluate fragmented and condensed unclear chromatins after treatment with **1** (10 nM) for 3h, **8** (20 nM) or cisplatin (33 nM) for 24h.
