# Supplementary material for: Steroidal Glycosides from Convallaria majalis Whole Plants and Their Cytotoxic Activity
Source: Int J Mol Sci. 2017 Nov 7;18(11):2358. doi: 10.3390/ijms18112358 (PMC5713327; doi:10.3390/ijms18112358)

Steroidal glycosides from *Convallaria majalis* whole plants and Their Cytotoxic Activity

Yukiko Matsuo, Daisuke Shinoda, Aina Nakamaru, Kuni Kamohara, Hiroshi Sakagami, and Yoshihiro Mimaki

Supplementary Materials

Fig. 1.2. ^{1}H and ^{13}C NMR spectral for **4**

Fig. 3.4 HMBC and MS spectrometry for **4**

Fig. 5.6. ^{1}H and ^{13}C NMR spectral for **5**

Fig. 7.8 HMBC and MS spectrometry for **5**

Fig. 9.10. ^{1}H and ^{13}C NMR spectral for **6**

Fig. 11.12 HMBC and MS spectrometry for **6**

Fig. 13.14. ^{1}H and ^{13}C NMR spectral for **10**

Fig. 15.16 HMBC and MS spectrometry for **10**

Fig. 17.18. ^{1}H and ^{13}C NMR spectral for **11**

Fig. 19.20 HMBC and MS spectrometry for **11**

Fig. 21.22. ^{1}H and ^{13}C NMR spectral for **12**

Fig. 23.24 HMBC and MS spectrometry for **12**

Fig. 25.26. ^{1}H and ^{13}C NMR spectral for **13**

Fig. 27.28 HMBC and MS spectrometry for **13**

Fig. 29.30. ^{1}H and ^{13}C NMR spectral for **13a**

Fig. 31.32 HMBC and MS spectrometry for **13a**

Fig. 33.34. ^{1}H and ^{13}C NMR spectral for **14**

Fig. 35.36 HMBC and MS spectrometry for **14**

Fig. 37.38. ^{1}H and ^{13}C NMR spectral for **15**

Fig. 39.40 HMBC and MS spectrometry for **15**

Fig.41. Toxicity curves of **1** and **8.**

Fig. 1.2. ^{1}H and ^{13}C NMR spectral for **4**

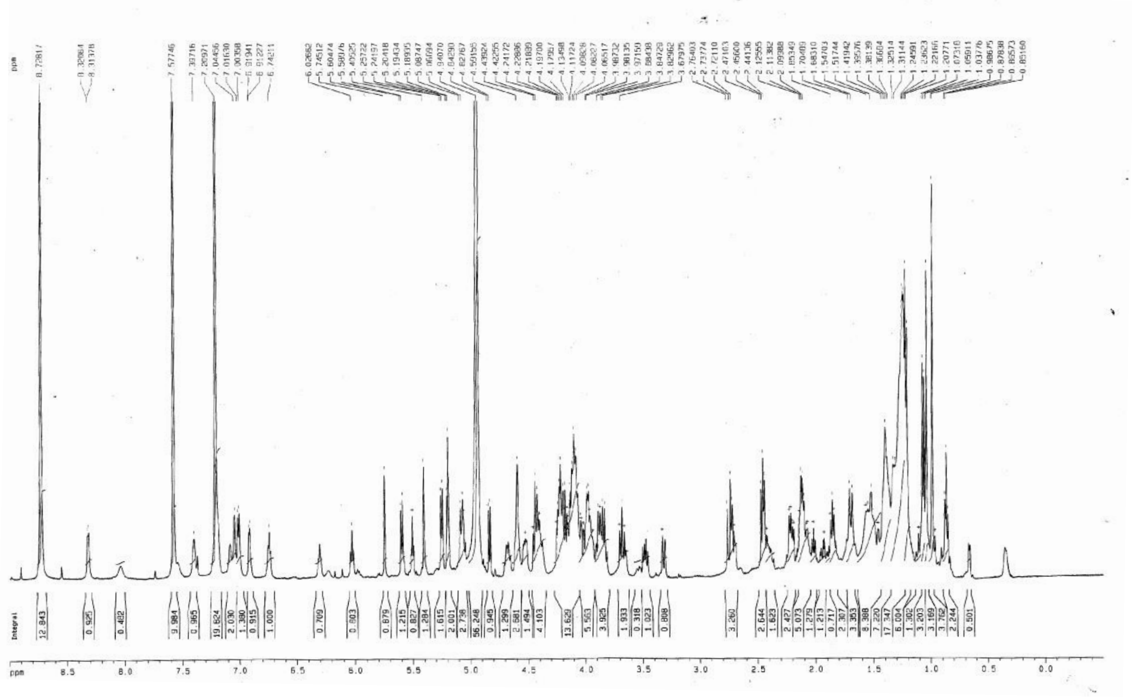

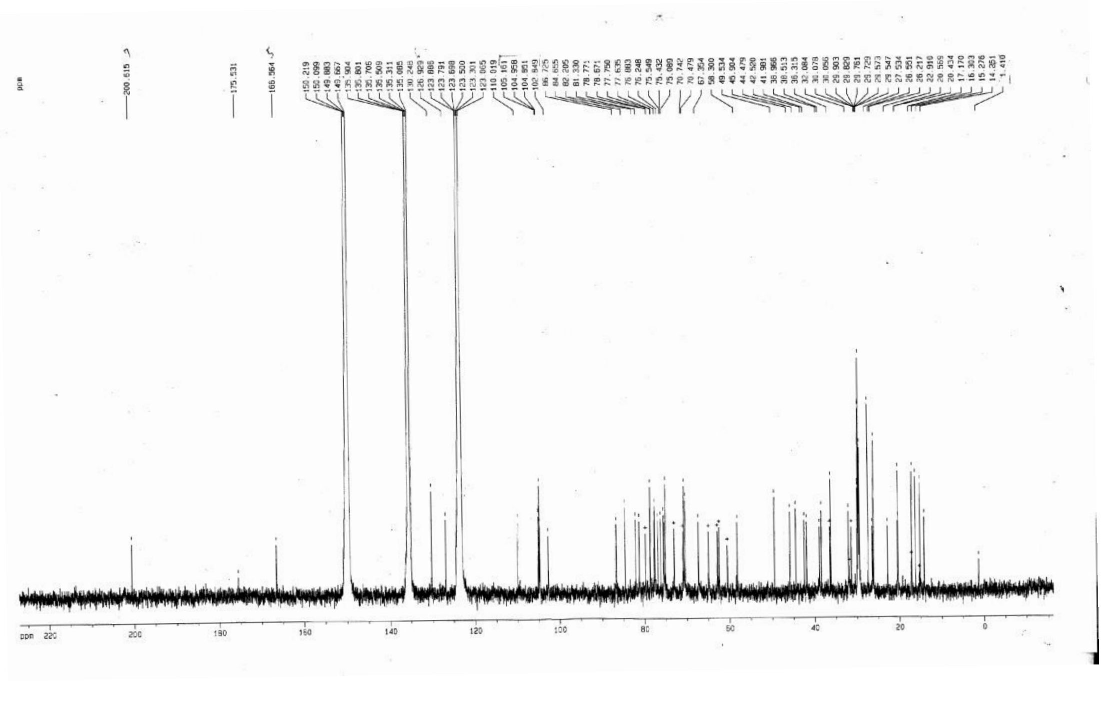

Fig. 3.4. HMBC and MS spectrometry for **4**

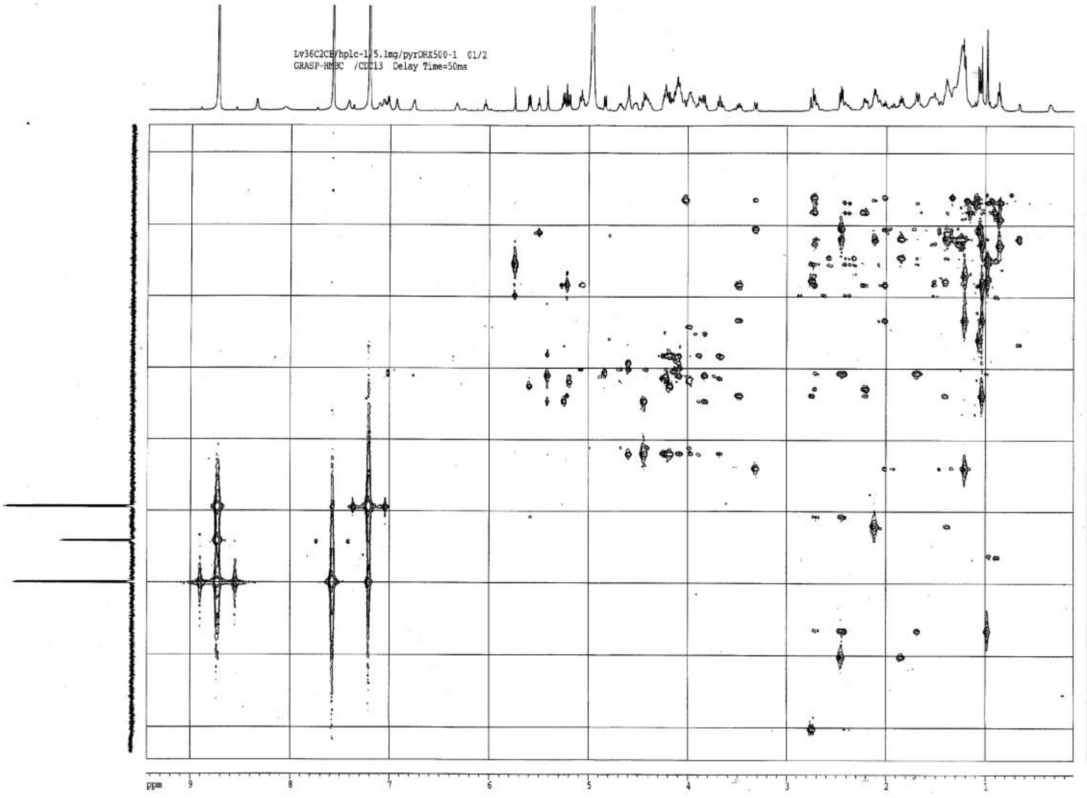

Elemental Composition Report **Page 1**

Single Mass Analysis
Tolerance = 10.0 PPM / DBE: min = -1.5, max = 60.0
Element prediction: Off
Number of isotope peaks used for i-FIT = 3

Monoisotopic Mass, Even Electron Ions
17 formula(e) evaluated with 2 results within limits (all results (up to 1000) for each mass)
Elements Used:
C: 20-80 H: 30-80 O: 23-24 Na: 0-1
LV36C2CEH1
SHINODA 001 104 (1.964) AM (Cen,4, 80.00, Ar,0.0,0.00,0.70); Sm (SG, 1x3.00); Cm (99:107) TOF MS ES+ 1.89e+004

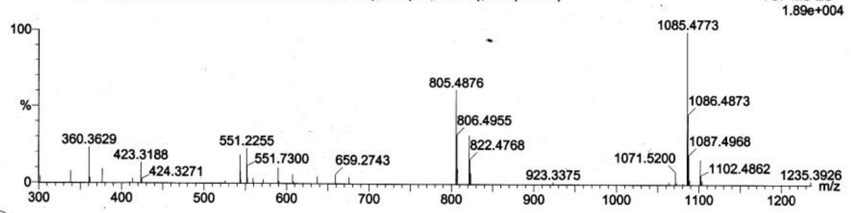

Minimum:				-1.5		
Maximum:		5.0	10.0	60.0		
Mass	Calc. Mass	mDa	PPM	DBE	i-FIT	Formula
1085.4773	1085.4781	-0.8	-0.7	11.5	179.3	C50 H78 O24 Na
	1085.4805	-3.2	-2.9	14.5	245.2	C52 H77 O24

Fig. 5.6. ^{1}H and ^{13}C NMR spectral for **5**

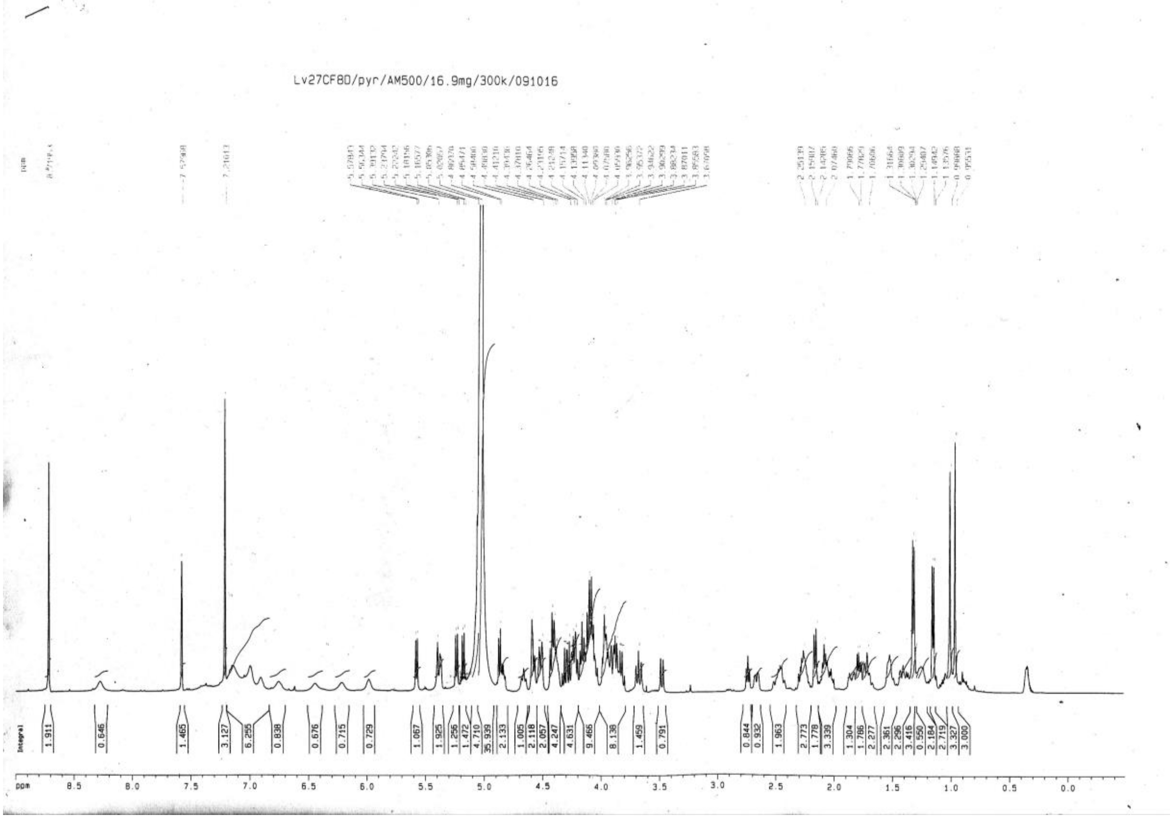

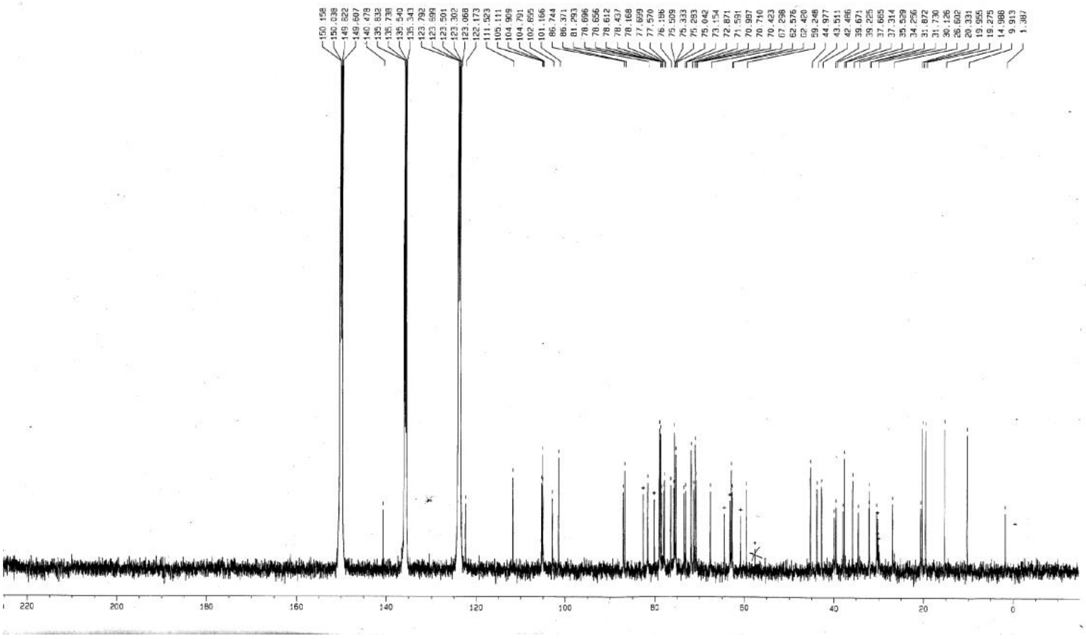

Fig. 7.8. HMBC and MS spectrometry for **5**

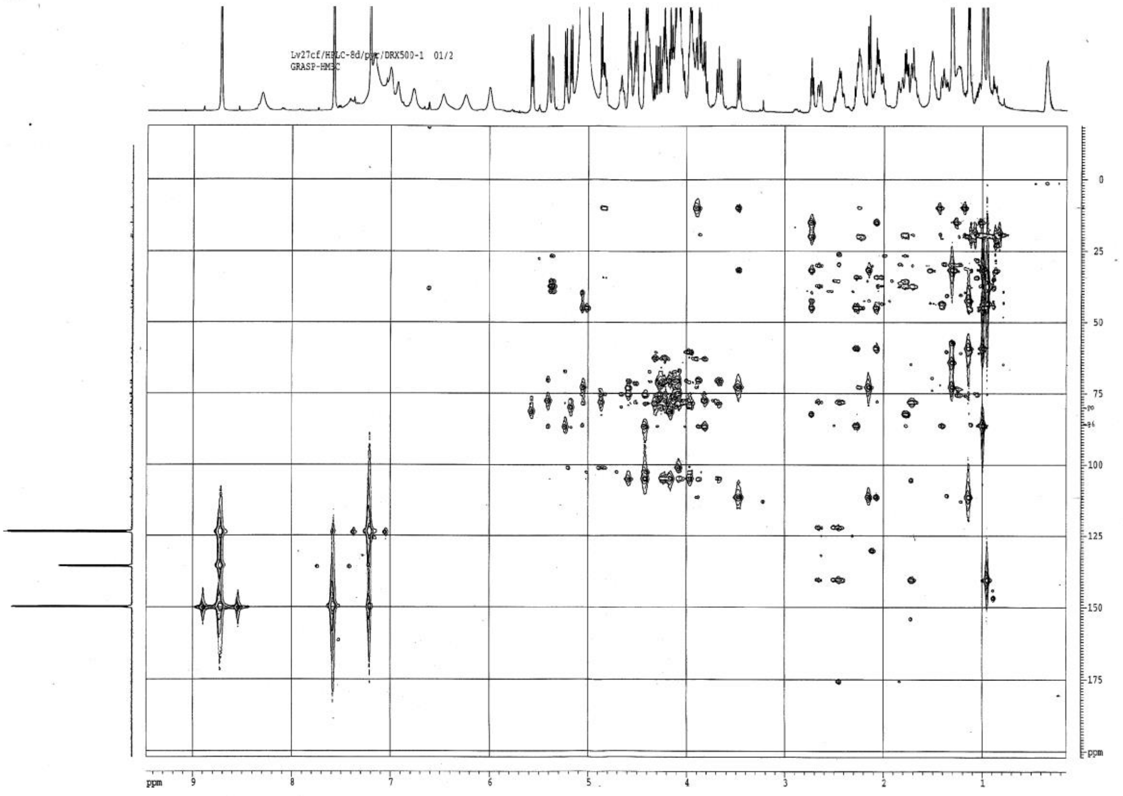

Elemental Composition Report **Page 1**

Single Mass Analysis
Tolerance = 10.0 PPM / DBE: min = -1.5, max = 60.0
Element prediction: Off
Number of isotope peaks used for i-FIT = 3

Monoisotopic Mass, Even Electron Ions
143 formula(e) evaluated with 5 results within limits (all results (up to 1000) for each mass)
Elements Used:
C: 20-80 H: 30-100 B: 0-1 O: 27-29 Na: 0-1 I: 0-1

LV 27CF8D
SHINODA 001 24 (0.460) AM (Cen,4, 80.00, Ar,0.0,0.00,0.70); Sm (SG, 1x3.00); Cm (21:32)

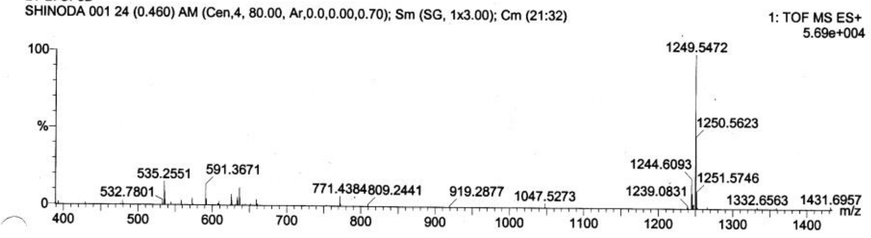

Minimum:				-1.5		
Maximum:		5.0	10.0	60.0		
Mass	Calc. Mass	mDa	PPM	DBE	i-FIT	Formula
1249.5472	1249.5465	0.7	0.6	11.5	3993.0	C56 H90 O29 Na
	1249.5461	1.1	0.9	-1.5	1404.3	C47 H99 B O28 I
	1249.5490	-1.8	-1.4	14.5	4548.9	C58 H89 O29
	1249.5450	2.2	1.8	18.5	4046.8	C60 H86 B O27
	1249.5425	4.7	3.8	15.5	3556.8	C58 H87 B O27 Na

Fig. 9.10. ^{1}H and ^{13}C NMR spectral for **6**

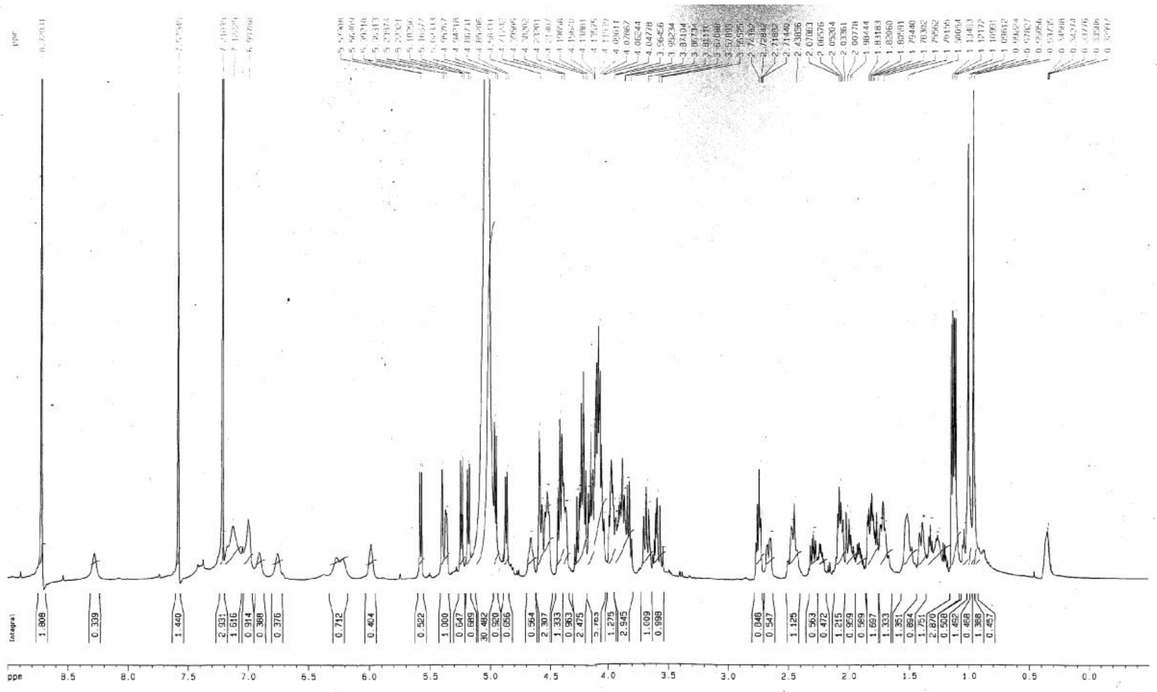

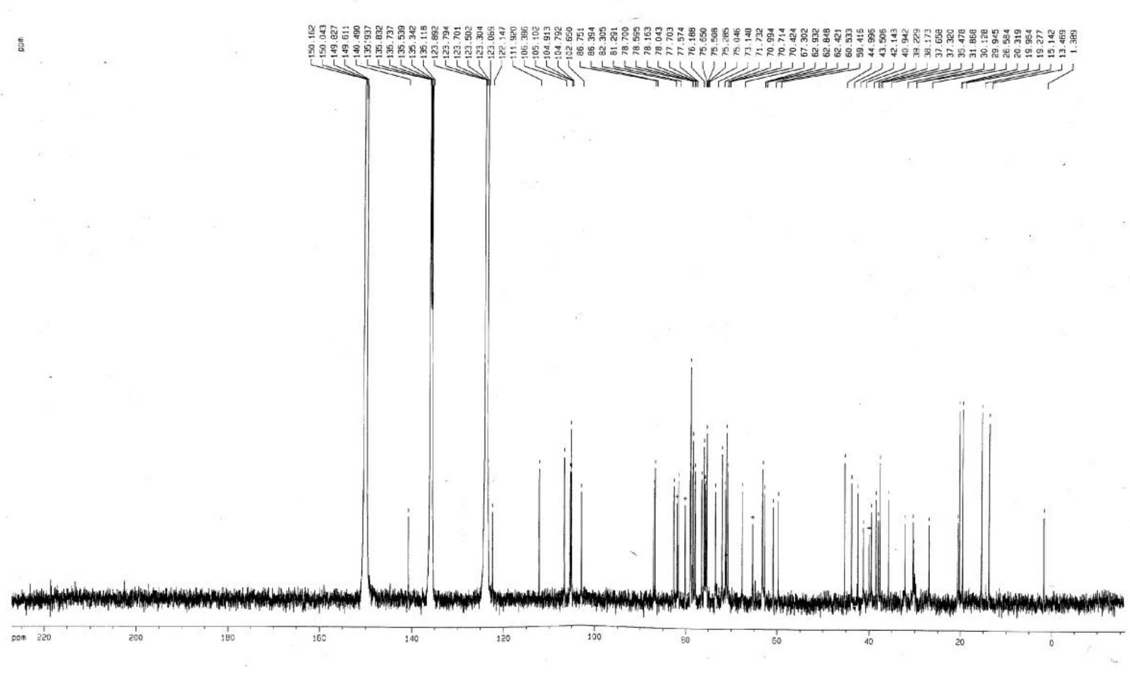

Fig. 11.12. HMBC and MS spectrometry for **6**

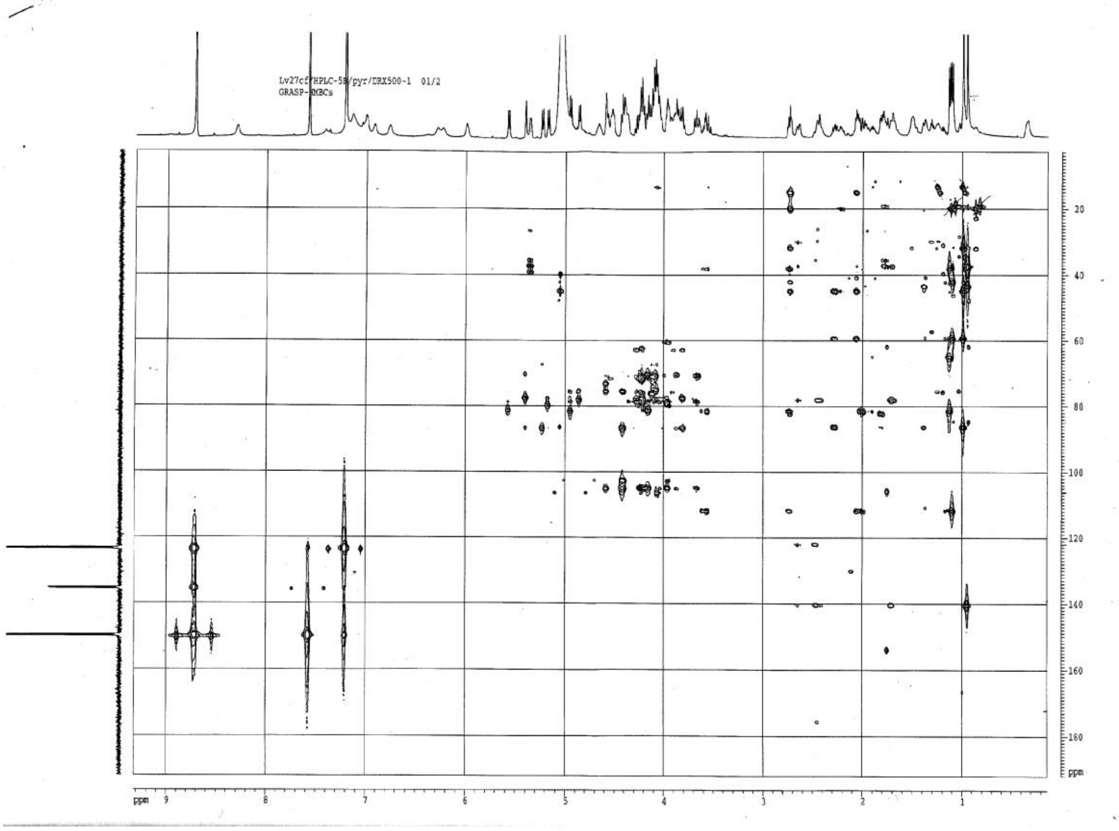

Elemental Composition Report **Page 1**

Single Mass Analysis
Tolerance = 20.0 PPM / DBE: min = -1.5, max = 40.0
Element prediction: Off
Number of isotope peaks used for i-FIT = 3

Monoisotopic Mass, Even Electron Ions
84 formula(e) evaluated with 6 results within limits (all results (up to 1000) for each mass)
Elements Used:
C: 0-100 H: 0-100 O: 25-29 Na: 0-1
LV 27CF5B2
SHINODA001 72 (1.361) AM (Cen,4, 80.00, Ht,0.0,0.00,0.70); Sm (SG, 1x3.00); Cm (63:72) 1: TOF MS ES+
2.20e+003

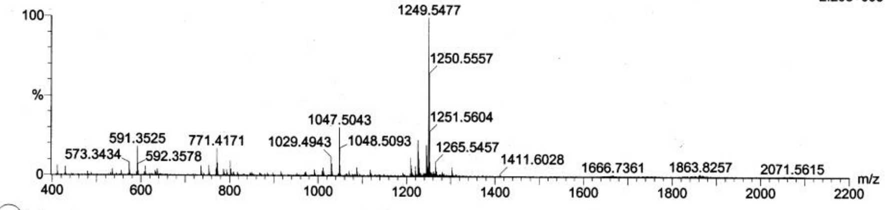

Minimum:				-1.5		
Maximum:		5.0	20.0	40.0		
Mass	Calc. Mass	mDa	PPM	DBE	i-FIT	Formula
1249.5477	1249.5490	-1.3	-1.0	14.5	0.5	C58 H89 O29
	1249.5465	1.2	1.0	11.5	1.0	C56 H90 O29 Na
	1249.5618	-14.1	-11.3	15.5	2.7	C60 H90 O26 Na
	1249.5254	22.3	17.8	16.5	5.6	C59 H86 O27 Na
	1249.5642	-16.5	-13.2	18.5	5.7	C62 H89 O26
	1249.5278	19.9	15.9	19.5	6.6	C61 H85 O27

Fig. 13.14. ^{1}H and ^{13}C NMR spectral for **10**

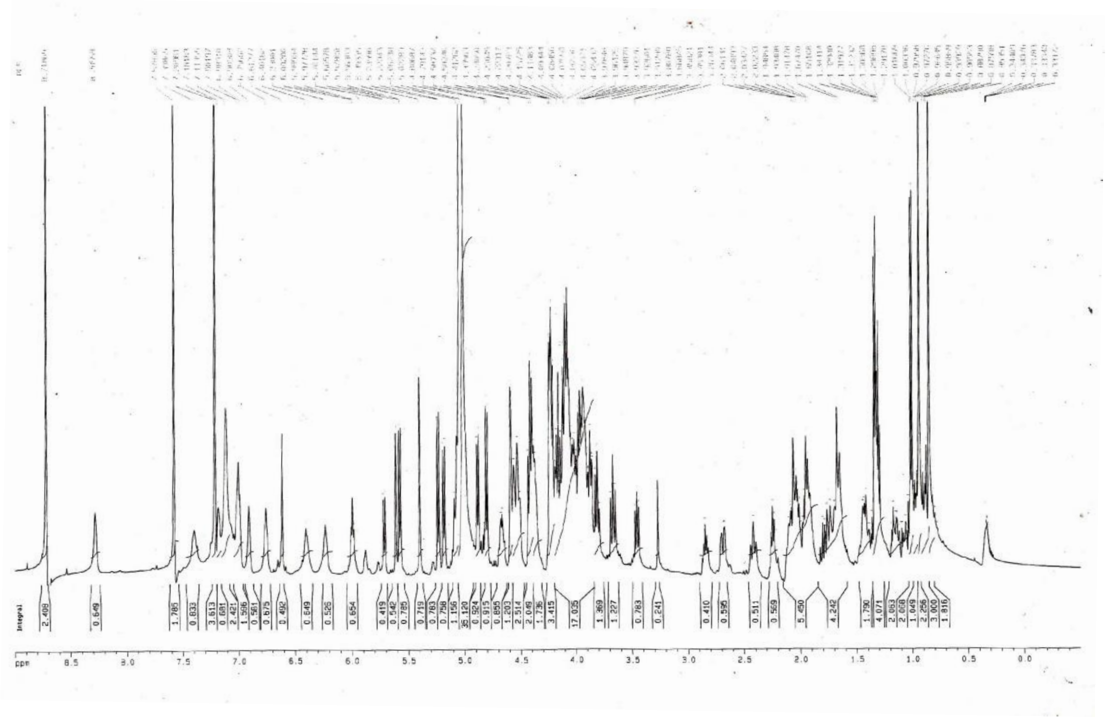

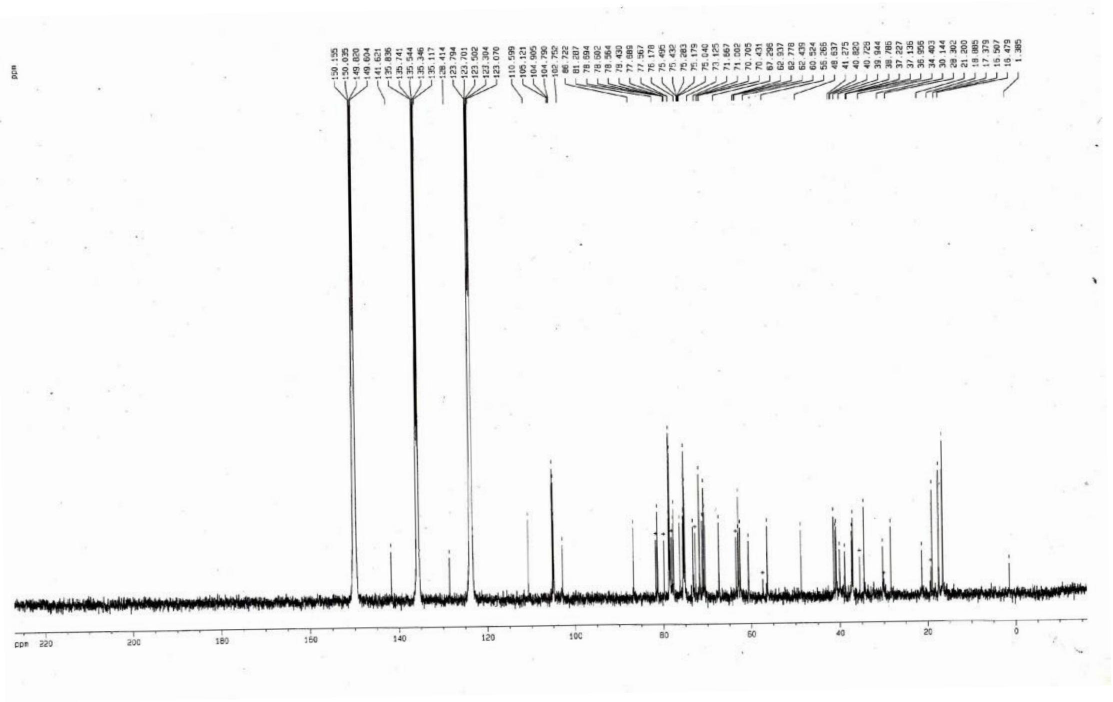

Fig. 15.16. HMBC and MS spectrometry for **10**

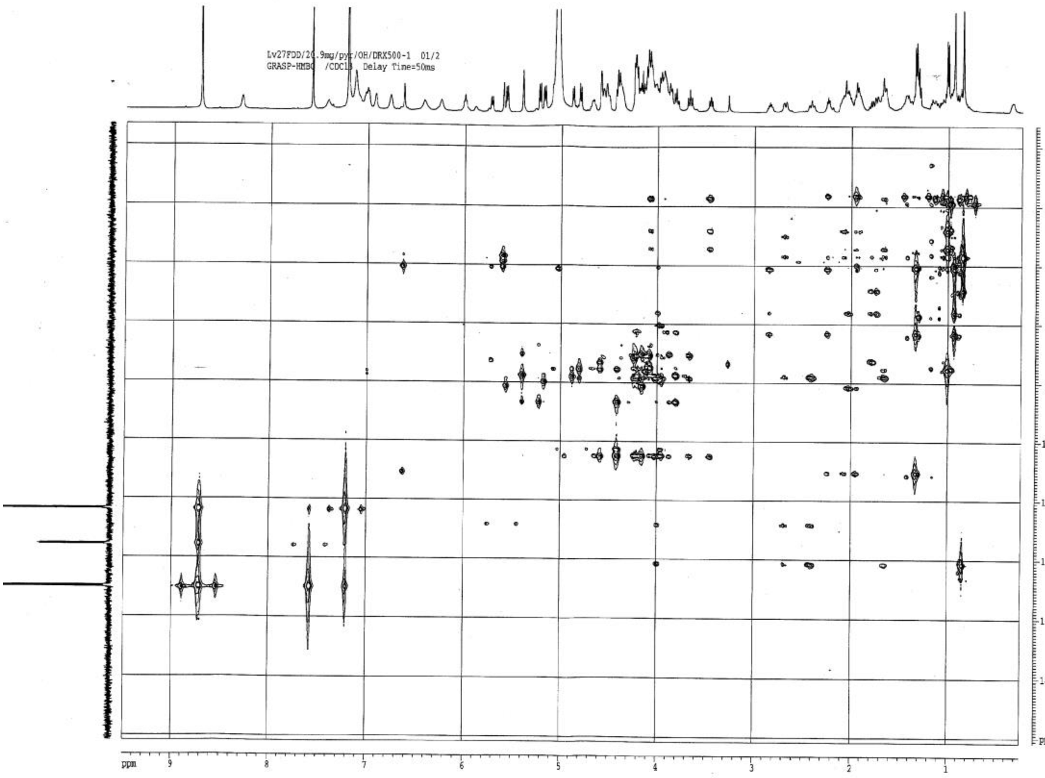

Elemental Composition Report **Page 1**

Single Mass Analysis
Tolerance = 20.0 PPM / DBE: min = -1.5, max = 300.0
Element prediction: Off
Number of isotope peaks used for i-FIT = 3

Monoisotopic Mass, Even Electron Ions
10 formula(e) evaluated with 1 results within limits (up to 50 closest results for each mass)
Elements Used:
C: 1-300 H: 1-1000 O: 29-29 Na: 1-1
Lv27FDD2
M-11032 101 (2.115) AM2 (Ar,22000.0,0.00,0.00); ABS; Cm (98:112) 1: TOF MS ES+
1.48e+007

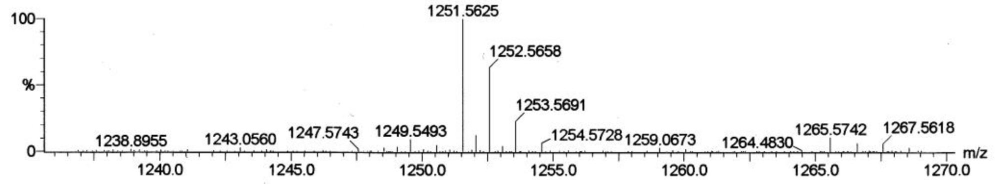

Minimum:				-1.5				
Maximum:		100.0	20.0	300.0				
Mass	Calc. Mass	mDa	PPM	DBE	i-FIT	Norm	Conf(%)	Formula
1251.5625	1251.5622	0.3	0.2	10.5	286.1	n/a	n/a	C56 H92 O29 Na

Fig. 17.18. ^{1}H and ^{13}C NMR spectral for **11**

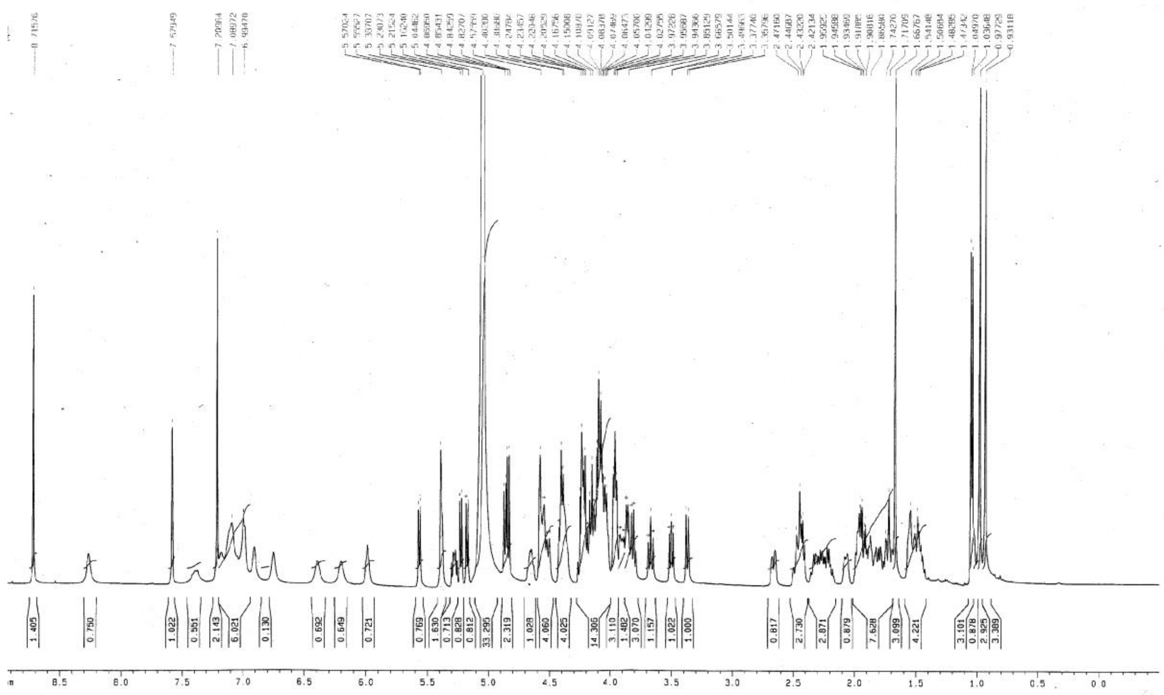

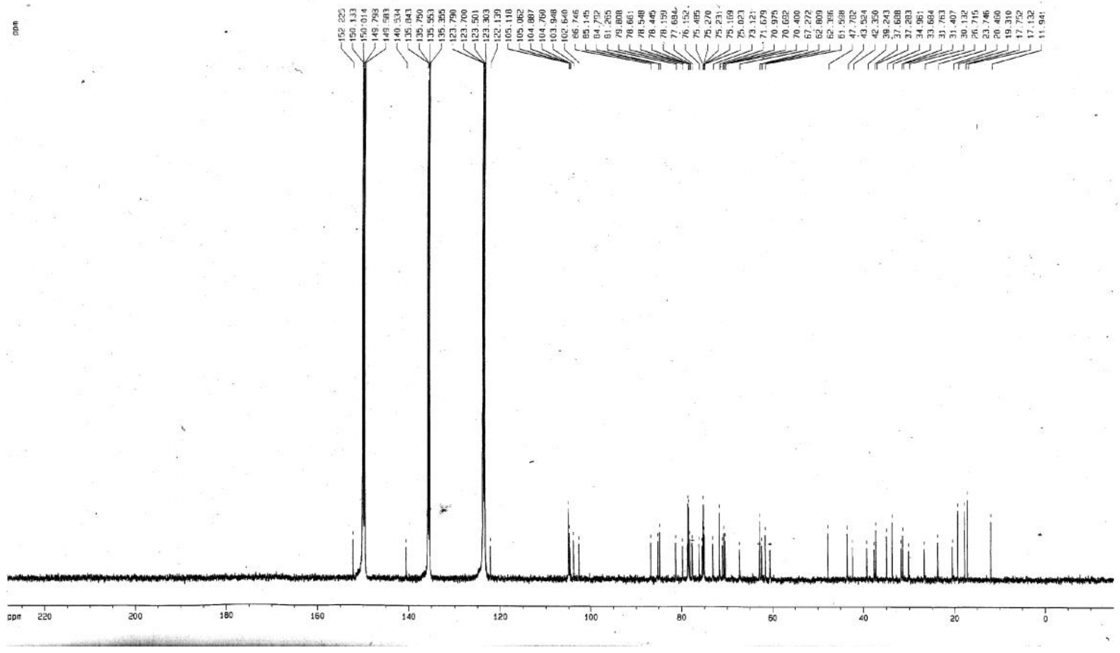

Fig. 19.20 HMBC and MS spectrometry for **11**

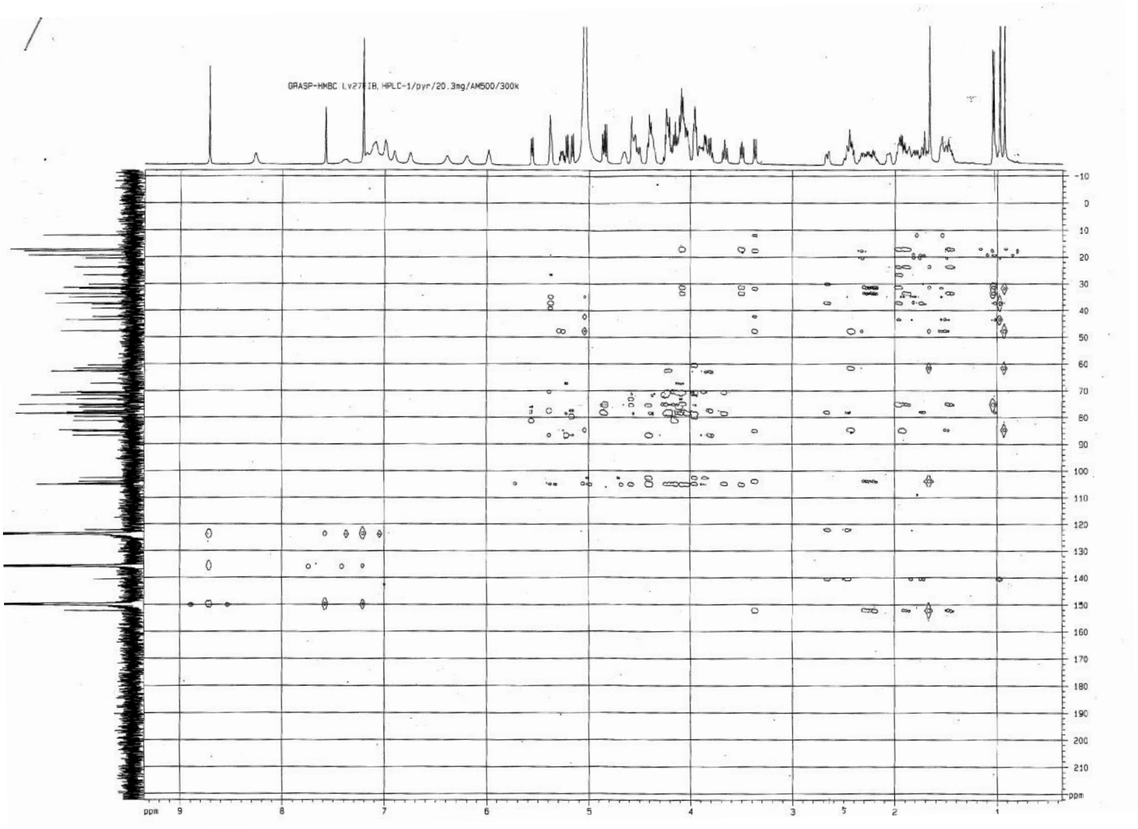

Elemental Composition Report **Page 1**

Single Mass Analysis (displaying only valid results)
Tolerance = 6.0 PPM / DBE: min = -1.5, max = 40.0
Isotope cluster parameters: Separation = 1.0 Abundance = 1.0%

Monoisotopic Mass, Odd and Even Electron Ions
45 formula(e) evaluated with 2 results within limits (all results (up to 1000) for each mass)

LV27FIBH11 HPLC-1 23-Oct-2009
SHINODA 002 23 (0.424) AM (Cen,3, 80.00, Ht,5000.0,0.00,0.80); Sm (SG, 2x3.00); Cm (3:24) 1: TOF MS ES+

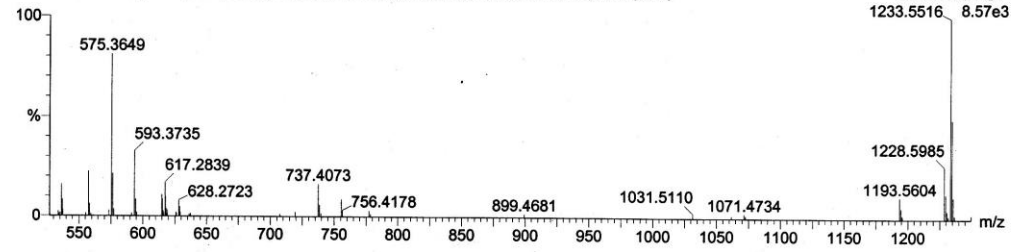

Minimum:				-1.5		
Maximum:		10.0	6.0	40.0		
Mass	Calc. Mass	mDa	PPM	DBE	Score	Formula
1233.5516	1233.5516	0.0	0.0	11.5	1	C56 H90 O28 Na
	1233.5540	-2.4	-1.9	14.5	2	C58 H89 O28

Fig. 21.22. ^{1}H and ^{13}C NMR spectral for **12**

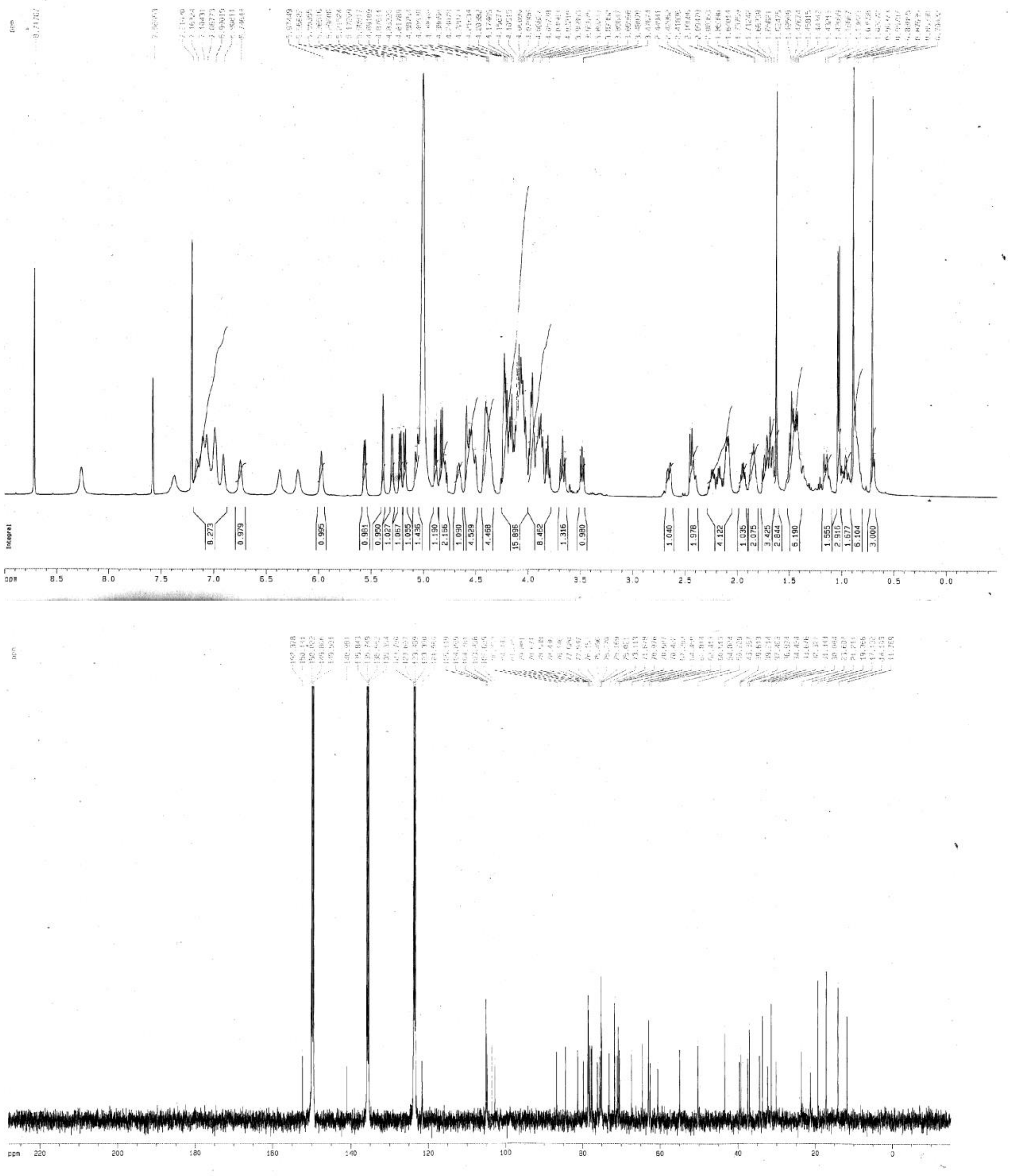

Fig. 23.24. HMBC and MS spectrometry for **12**

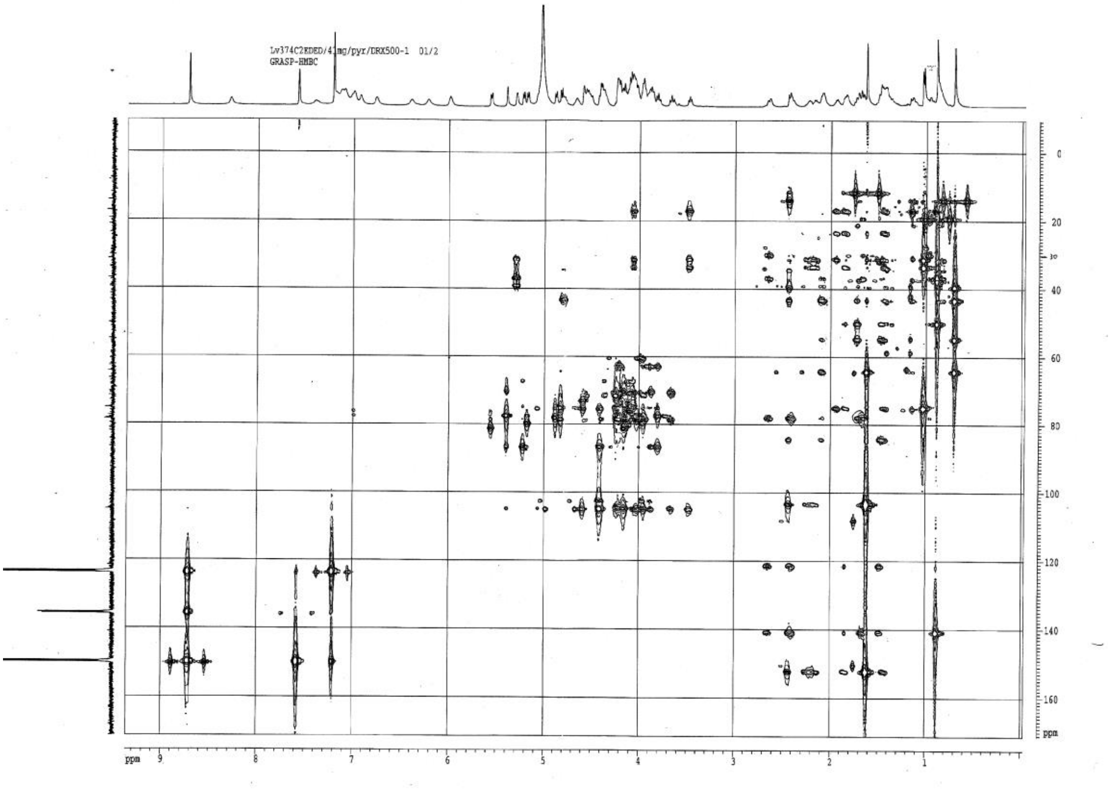

Elemental Composition Report **Page 1**

Single Mass Analysis
Tolerance = 20.0 PPM / DBE: min = -1.5, max = 300.0
Element prediction: Off
Number of isotope peaks used for i-FIT = 3

Monoisotopic Mass, Even Electron Ions
10 formula(e) evaluated with 1 results within limits (up to 50 closest results for each mass)
Elements Used:
C: 1-300 H: 1-1000 O: 27-27
Lv374C2ED
M-11030 167 (3.483) AM2 (Ar,22000.0,0.00,0.00); ABS; Cm (156:170) 1: TOF MS ES+ 3.31e+007

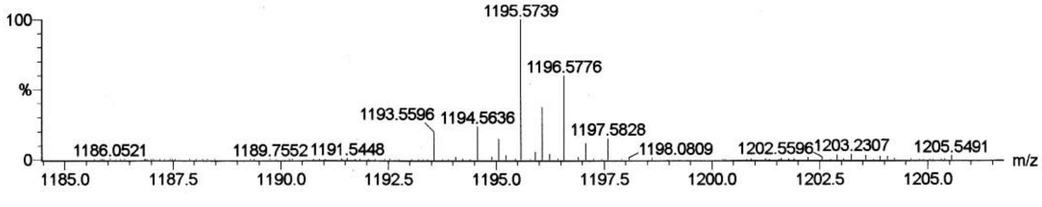

	Calc. Mass	mDa	PPM	DBE				
Minimum:				-1.5				
Maximum:		100.0	20.0	300.0				

Mass	Calc. Mass	mDa	PPM	DBE	i-FIT	Norm	Conf(%)	Formula
1195.5739	1195.5748	-0.9	-0.8	11.5	295.0	n/a	n/a	C56 H91 O27

Fig. 25.26. ^{1}H and ^{13}C NMR spectral for **13**

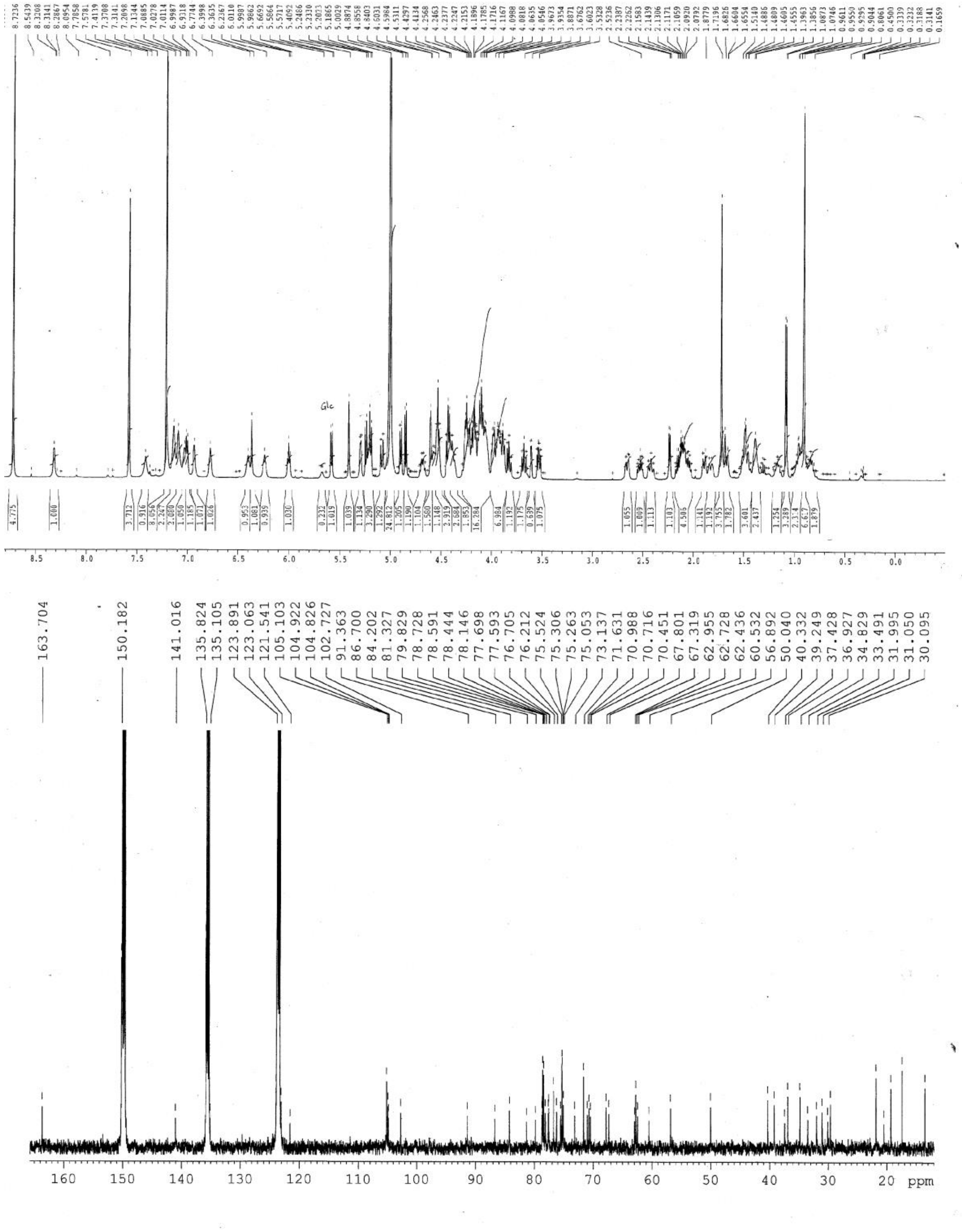

Fig. 27.28. HMBC and MS spectrometry for **13**

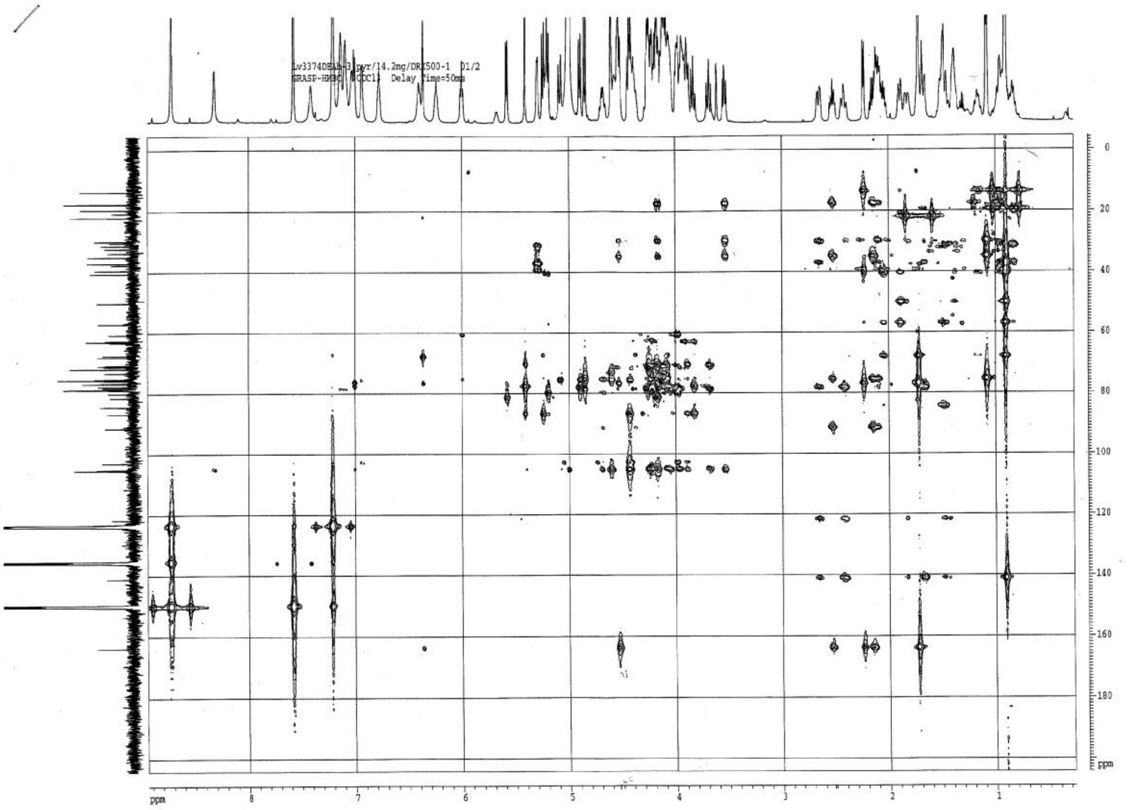

Elemental Composition Report **Page 1**

Single Mass Analysis
Tolerance = 10.0 PPM / DBE: min = -1.5, max = 60.0
Element prediction: Off
Number of isotope peaks used for i-FIT = 3

Monoisotopic Mass, Even Electron Ions
110 formula(e) evaluated with 2 results within limits (all results (up to 1000) for each mass)
Elements Used:
C: 20-90 H: 30-140 O: 25-30 Na: 0-1
374DEAH3
SHINODAI 001 39 (0.742) AM (Cen,4, 80.00, Ar,0.0,0.00,0.70); Sm (SG, 1x3.00); Cm (24:43)

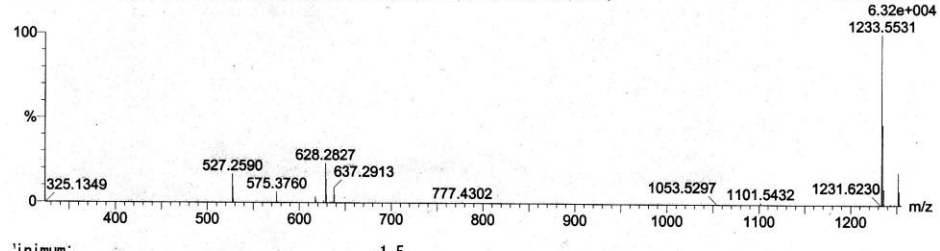

Minimum:				-1.5		
Maximum:		5.0	10.0	60.0		
Mass	Calc. Mass	mDa	PPM	DBE	i-FIT	Formula
1233.5531	1233.5540	-0.9	-0.7	14.5	6476.6	C58 H89 O28
	1233.5516	1.5	1.2	11.5	5777.0	C56 H90 O28 Na

Fig. 29.30. ^{1}H and ^{13}C NMR spectral for **13a**

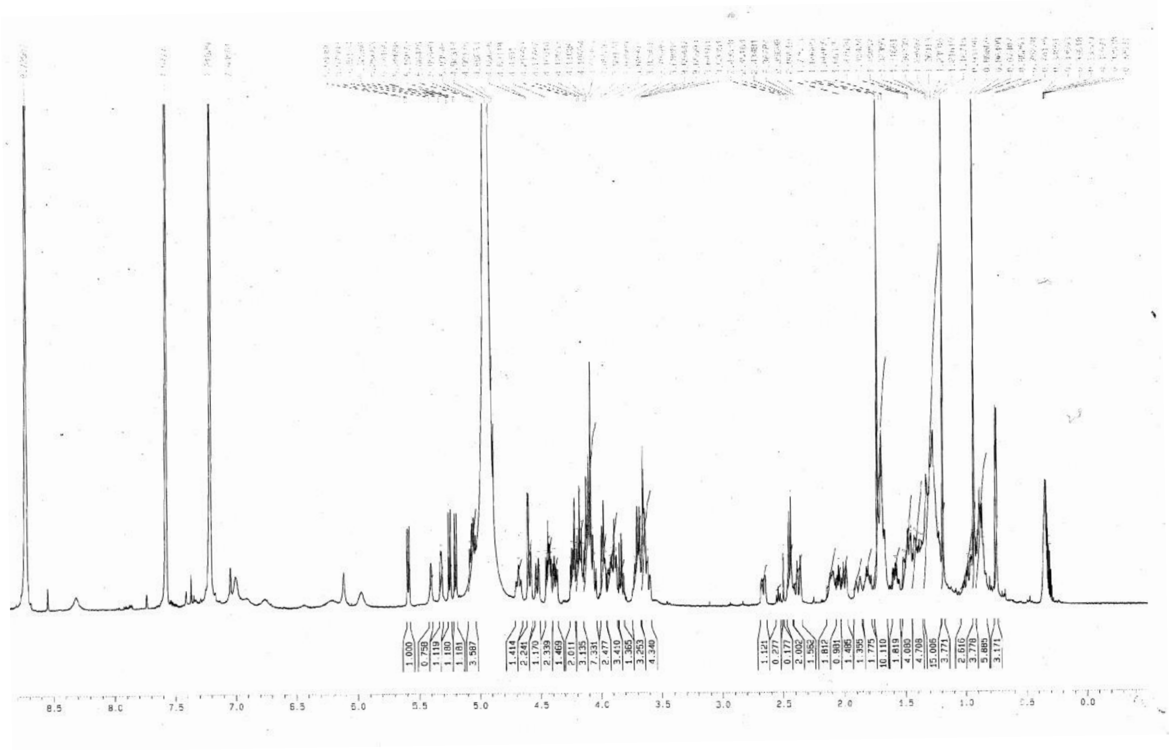

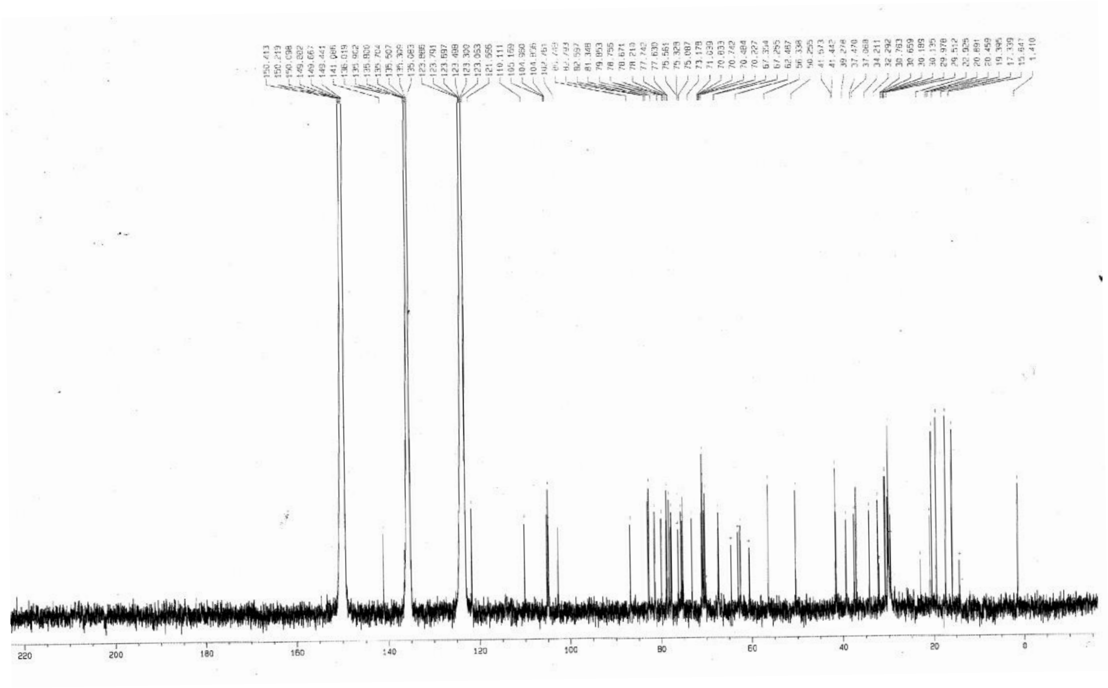

Fig. 31.32 HMBC and MS spectrometry for **13a**

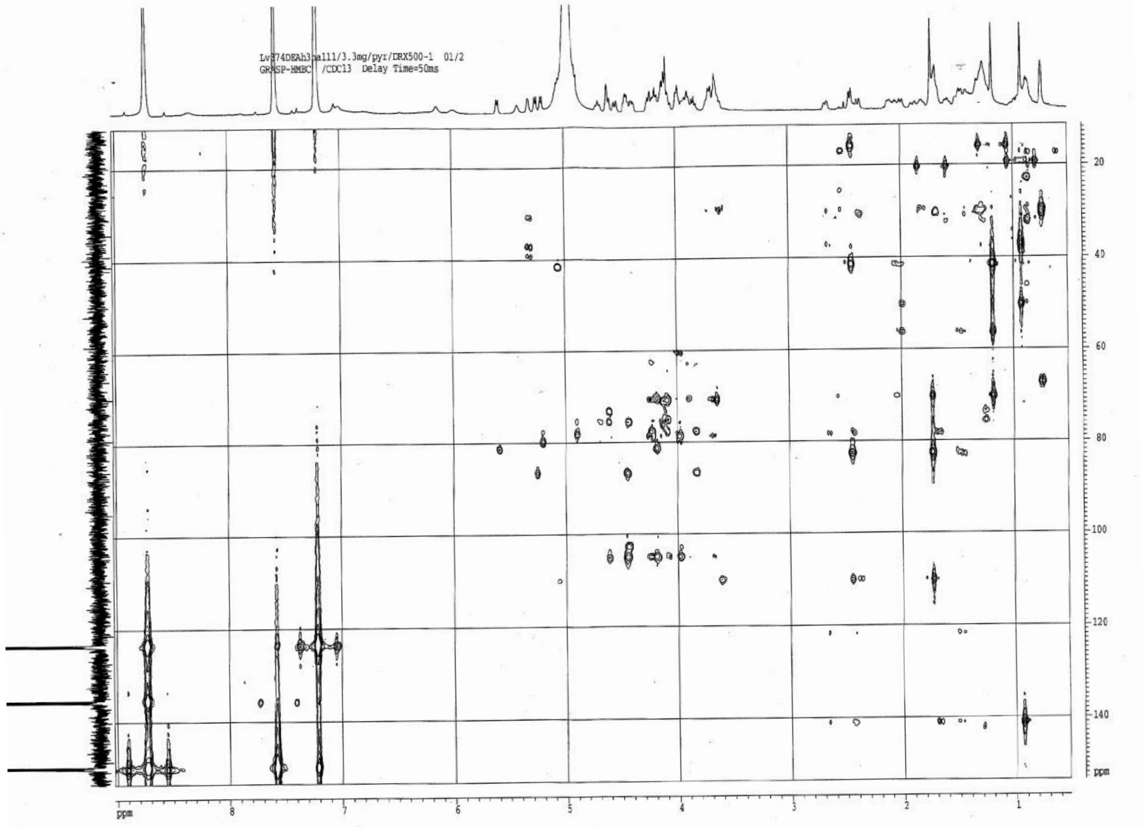

Elemental Composition Report **Page 1**

Single Mass Analysis
Tolerance = 15.0 PPM / DBE: min = -1.5, max = 60.0
Element prediction: Off
Number of isotope peaks used for i-FIT = 3

Monoisotopic Mass, Even Electron Ions
53 formula(e) evaluated with 3 results within limits (all results (up to 1000) for each mass)
Elements Used:
C: 10-100 H: 10-100 O: 17-23 Na: 1-1
LV374111
SHINODA 001 207 (3.902) AM (Cen,4, 80.00, Ar,0.0,0.00,0.70); Sm (SG, 1x3.00); Cm (207:232)

1: TOF MS ES+
5.37e+004

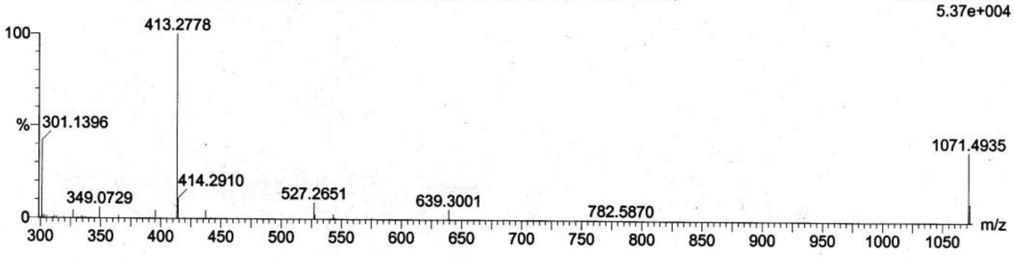

Minimum:				-1.5		
Maximum:		5.0	15.0	60.0		
Mass	Calc. Mass	mDa	PPM	DBE	i-FIT	Formula
1071.4935	1071.4929	0.6	0.6	19.5	10172.7	C57 H76 O18 Na
	1071.4988	-5.3	-4.9	10.5	9722.1	C50 H80 O23 Na
	1071.4777	15.8	14.7	15.5	9939.4	C53 H76 O21 Na

Fig. 33.34. ^{1}H and ^{13}C NMR spectral for **14**

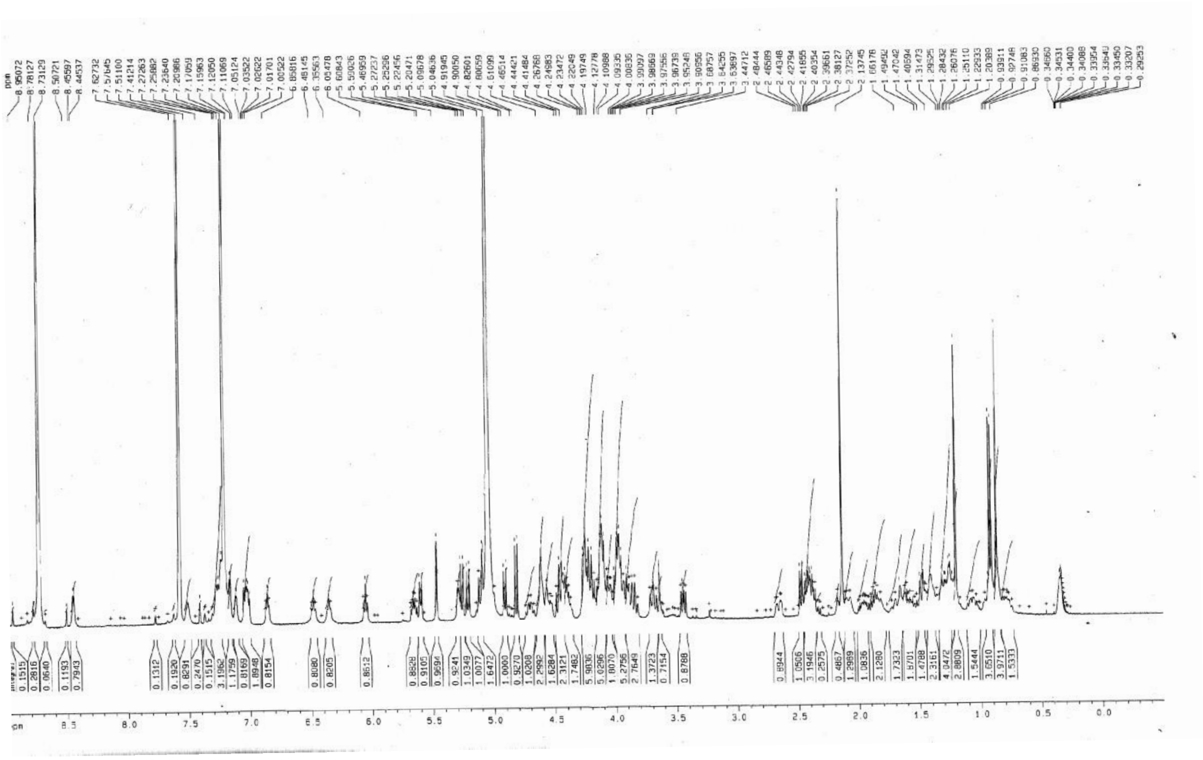

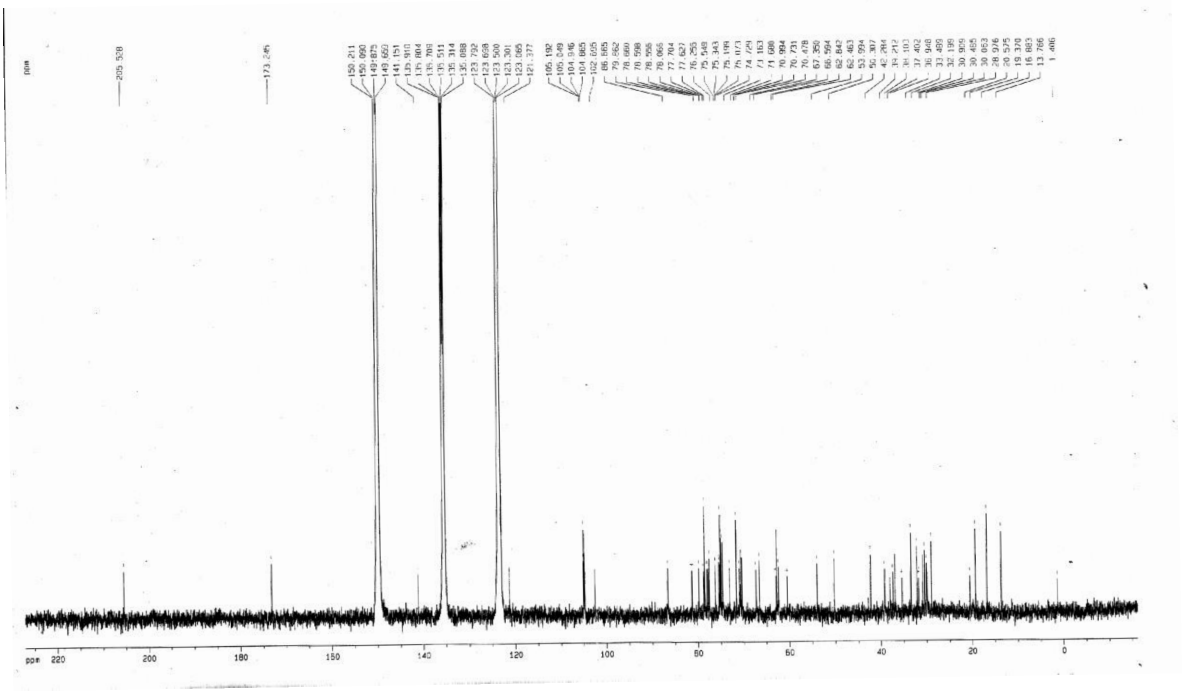

Fig. 35.36. HMBC and MS spectrometry for **14**

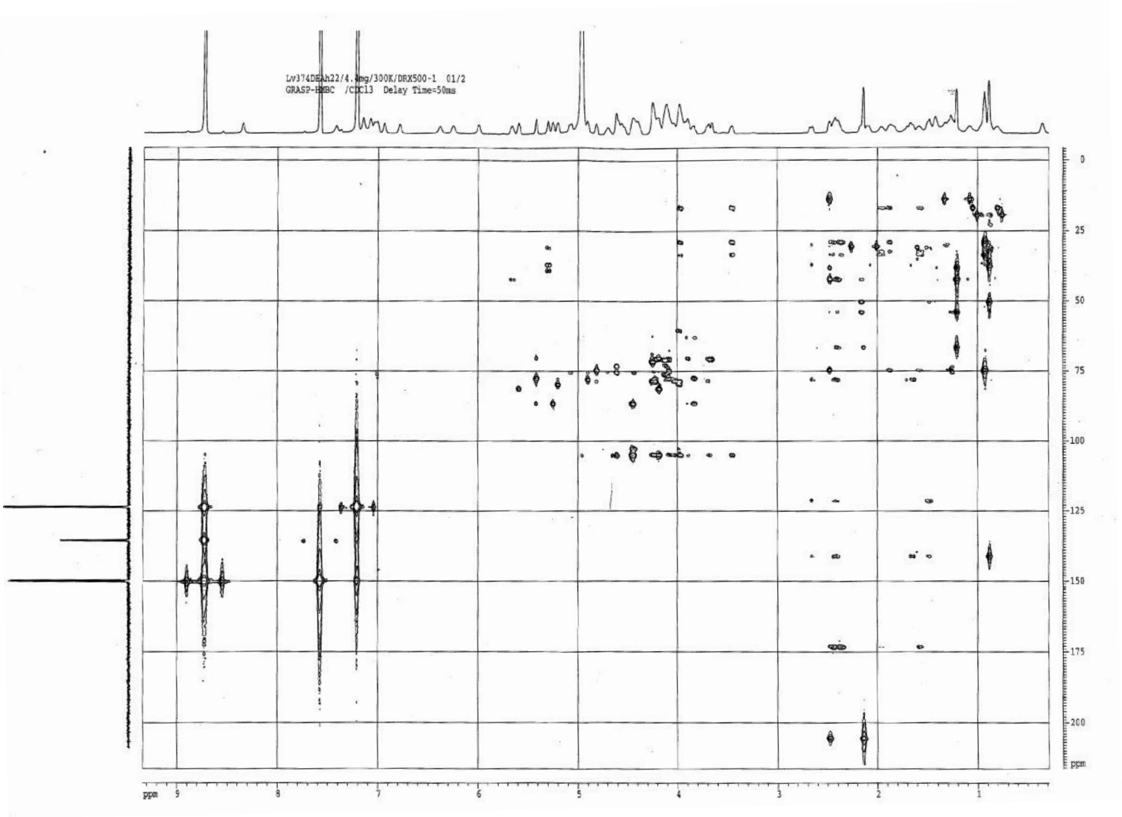

Elemental Composition Report **Page 1**

Single Mass Analysis
Tolerance = 20.0 PPM / DBE: min = -1.5, max = 300.0
Element prediction: Off
Number of isotope peaks used for i-FIT = 3

Monoisotopic Mass, Even Electron Ions
10 formula(e) evaluated with 1 results within limits (up to 50 closest results for each mass)
Elements Used:
C: 1-300 H: 1-1000 O: 29-29 Na: 1-1
74DEAH22
M-11031 129 (2.691) AM2 (Ar,22000.0,0.00,0.00); ABS; Cm (115:129) 1: TOF MS ES+ 1.21e+007

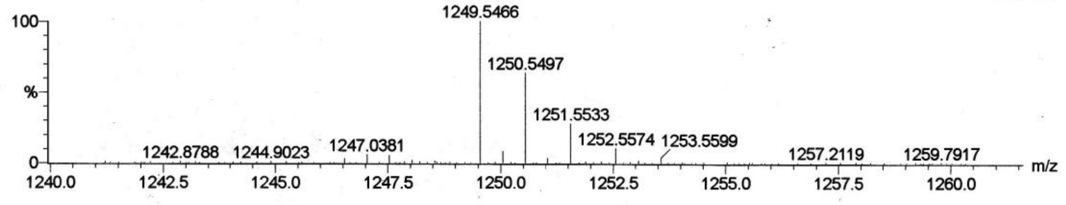

Minimum:				-1.5				
Maximum:		100.0	20.0	300.0				
Mass	Calc. Mass	mDa	PPM	DBE	i-FIT	Norm	Conf(%)	Formula
1249.5466	1249.5465	0.1	0.1	11.5	279.7	n/a	n/a	C56 H90 O29 Na

Fig. 37.38. ^{1}H and ^{13}C NMR spectral for **15**

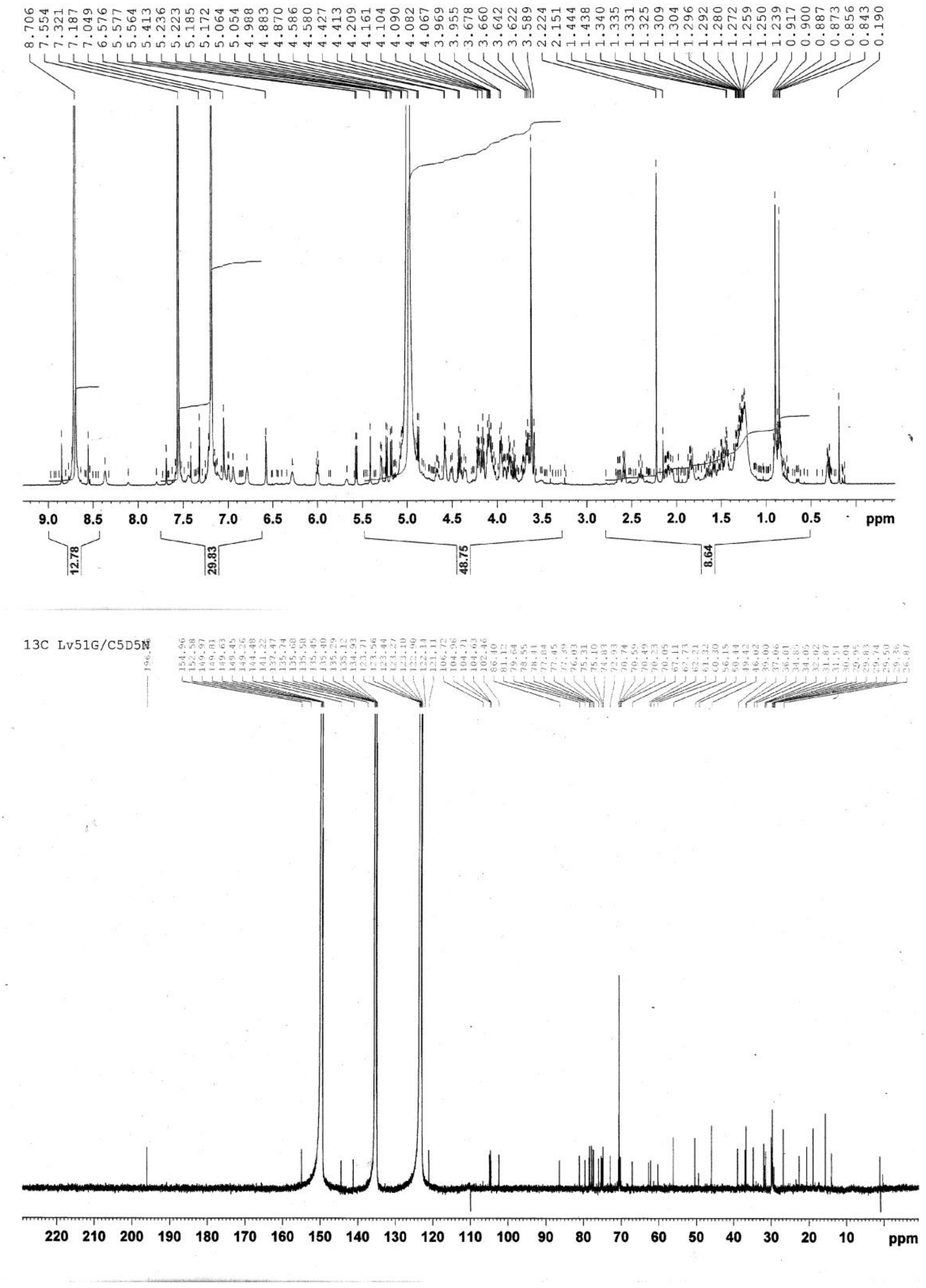

Fig. 39.40 HMBC and MS spectrometry for **15**

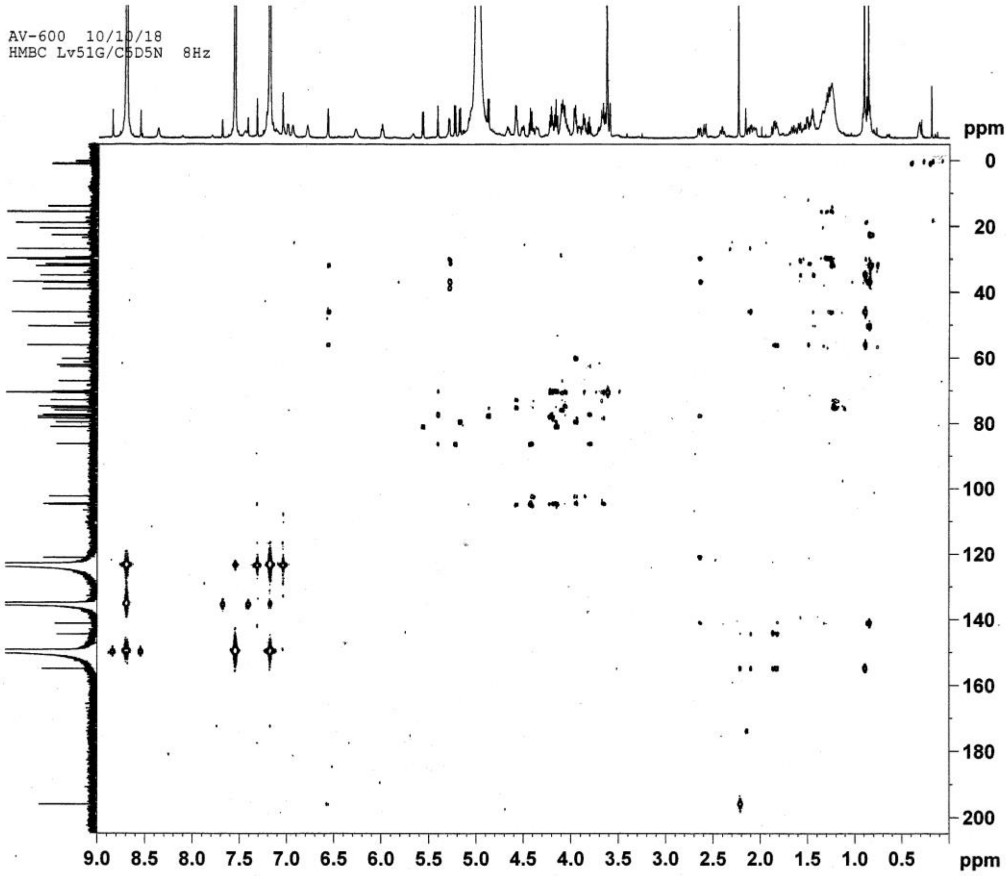

Elemental Composition Report **Page 1**

Single Mass Analysis
Tolerance = 15.0 PPM / DBE: min = -1.5, max = 60.0
Element prediction: Off
Number of isotope peaks used for i-FIT = 3

Monoisotopic Mass, Even Electron Ions
15 formula(e) evaluated with 1 results within limits (all results (up to 1000) for each mass)
Elements Used:
C: 10-100 H: 10-100 O: 20-21 Na: 1-1
LV51G
SHINODA 001 57 (1.080) AM (Cen,4, 80.00, Ar,0.0,0.00,0.70); Sm (SG, 1x3.00); Cm (54:71) 1: TOF MS ES+ 1.13e+005

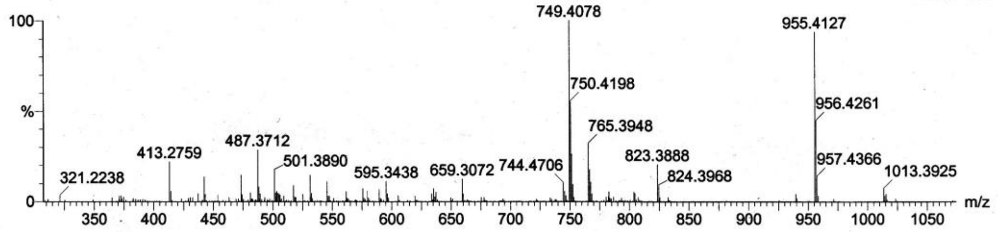

Minimum:				-1.5		
Maximum:		5.0	15.0	60.0		
Mass	Calc. Mass	mDa	PPM	DBE	i-FIT	Formula
955.4127	955.4151	-2.4	-2.5	10.5	160.2	C44 H68 O21 Na

Fig.41. Toxicity curves of **1** and **8.**

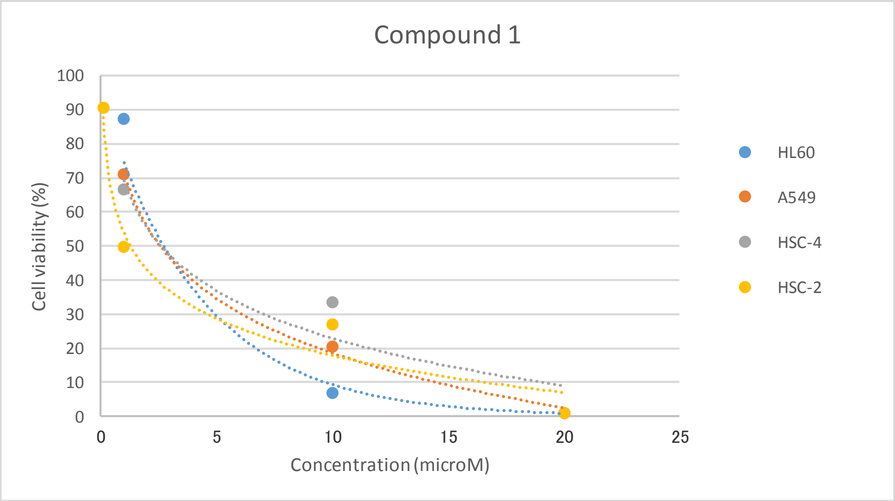

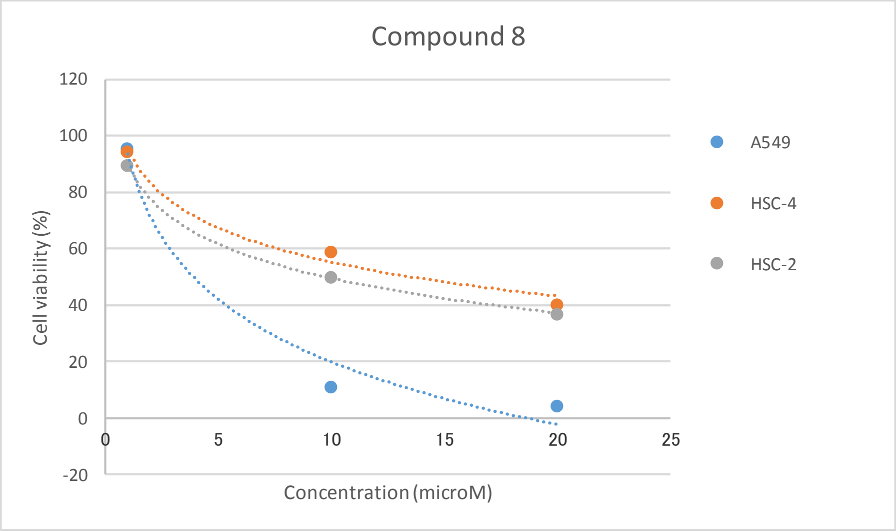

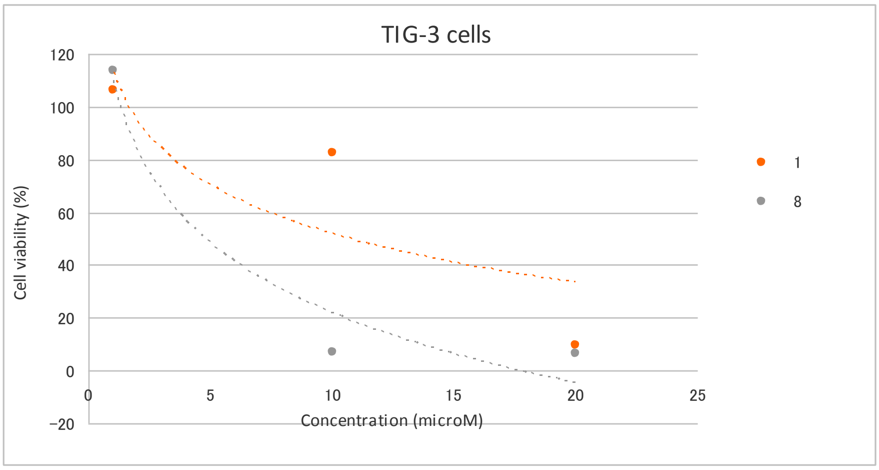

Supplement: Supplementary file 1 [file ijms-18-02358-s001.zip › Supplementary materials 2.pdf]
